# Multivariate Analysis Applied to Microwave-Driven Cyanide Polymerization: A Statistical View of a Complex System

**DOI:** 10.3390/polym15020410

**Published:** 2023-01-12

**Authors:** Cristina Pérez-Fernández, Elena González-Toril, Eva Mateo-Martí, Marta Ruiz-Bermejo

**Affiliations:** Centro de Astrobiología (CAB), CSIC-INTA, Ctra. Torrejón-Ajalvir, km 4, Torrejón de Ardoz, 28850 Madrid, Spain

**Keywords:** HCN-derived polymers, multivariate analysis, microwave-driven polymerization, hydrothermal systems

## Abstract

For the first time, chemometrics was applied to the recently reported microwave-driven cyanide polymerization. Fast, easy, robust, low-cost, and green-solvent processes are characteristic of these types of reactions. These economic and environmental benefits, originally inspired by the constraints imposed by plausible prebiotic synthetic conditions, have taken advantage of the development of a new generation of HCN-derived multifunctional materials. HCN-derived polymers present tunable properties by temperature and reaction time. However, the apparently random behavior observed in the evolution of cyanide polymerizations, assisted by microwave radiation over time at different temperatures, leads us to study this highly complex system using multivariate analytical tools to have a proper view of the system. Two components are sufficient to explain between 84 and 98% of the total variance in the data in all principal component analyses. In addition, two components explain more than 91% of the total variance in the data in the case of principal component analysis for categorical data. These consistent statistical results indicate that microwave-driven polymerization is a more robust process than conventional thermal syntheses but also that plausible prebiotic chemistry in alkaline subaerial environments could be more complex than in the aerial part of these systems, presenting a clear example of the “messy chemistry” approach of interest in the research about the origins of life. In addition, the methodology discussed herein could be useful for the data analysis of extraterrestrial samples and for the design of soft materials, in a feedback view between prebiotic chemistry and materials science.

## 1. Introduction

**Scheme 1:**
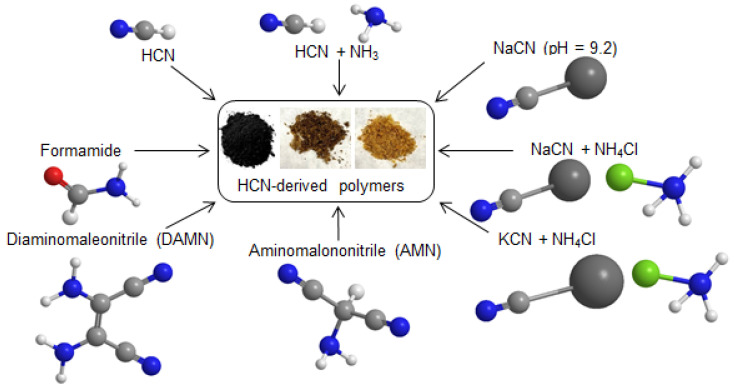
Brief view of the monomers/reactants used for the production of HCN-derived polymers. Note that the characteristics and properties of this particular kind of polymer are directly dependent on the synthetic conditions. For a detailed revision about the synthesis of the HCN-derived polymers, please see [6,29].

First, HCN oligomerization/polymerization has been appreciated in the field of prebiotic chemistry due to the first reported plausible abiotic synthesis of adenine from HCN and NH_3_ by Oró [1]. Later, HCN oligomerization/polymerization was considered a preferential route for the prebiotic production of purines and pyridines and other important bioorganics and related compounds (see, e.g., Refs. [2,3,4,5,6,7,8,9,10,11]) and as a paradigmatic system to understand the increasing molecular complexity from a simple one-carbon source (see, e.g., Refs. [12,13,14,15,16,17]). In addition, recently, HCN-derived polymers have received great attention in materials science [18,19]. Their tunable characteristics provide promising potential applications, such as coating materials of interest in biomedicine [19,20,21,22,23,24], as well as effective films against oxidation [25,26] and for the modification of electrodes for the development of biosensors [27]. Additionally, HCN-derived polymers have been tested as photocatalyzers [28], capacitors [29], semiconductors [30] and passive antimicrobial filters [31]. Moreover, they have been suggested as nanowires and ferroelectric materials [32] and as novel fillers for the generation of new nanocomposite materials [29]. Scheme 1 shows the main monomers used for the synthesis of this new kind of soft materials based on HCN.

Considering the properties found for HCN-derived polymers in the last few years, especially semiconductivity and photocatalysis, the prebiotic chemical space is currently significantly enriched [33]. This fact encourages the consideration of new approaches to discover protobiological reaction networks. In this way, HCN polymerizations must be considered beyond the traditional synthetic paths for the abiotic generation of biomonomers [29], presumably leading to the production of a new generation of multifunctional materials inspired by prebiotic chemistry, whose first examples are cited above. In addition, the synthetic methodologies for the production of HCN-derived polymers offer environmental and economic added value due to their robustness, low-cost, easy and green-solvent processes.

The reaction time and temperature are the main factors in the tunable characteristics of HCN-derived polymers [25,29,34,35,36]. Recently, microwave radiation (MWR) has been shown to significantly reduce the reaction time of NH_4_CN polymerization, producing cyanide polymers with spectroscopic and chemical compositional properties similar to the spectroscopic and chemical compositional properties of polymers synthesized using conventional thermal heating (CTH) but with morphological properties, size and shape that are very different [37]. The notable reduction in the reaction time and the generation of new nanoparticles and nanofibers of HCN-derived polymers [10,37], which have not previously been reported for these macromolecular systems, make microwave-driven cyanide polymerization highly attractive for comprehensive exploration. Moreover, this interest is dual. On the one hand, the use of a microwave reactor makes it possible to obtain new polymeric materials in truly faster synthetic processes and, on the other hand, aqueous synthesis at temperatures above 100 °C could be considered a simulation of natural hydrothermal systems, which attract great attention both in prebiotic chemistry and in astrobiology (see, e.g., Refs. [38,39,40,41,42,43]).

The exhaustive understanding of a chemical system involves knowing its kinetic history, since this history will condition the structural characteristics and properties of the final reaction products. Thus, the kinetics of the hydrothermal polymerizations of cyanide using CTH have been properly studied via gravimetric methodology. In these cases, the formation of the polymers synthesized at 80–90 °C followed an overall process that can be fitted to a Kamal–Sourour autocatalytic model [35], but at lower temperatures, 50–60 °C, these reactions were better adjusted to *n*th-order kinetic models [36]. In addition, the ultraviolet-visible (UV-Vis) spectra of the final raw solutions from all these cyanide polymerizations were also analyzed. The intensity relationships of representative absorption bands were properly fitted to Hill equation curves, indicating very complex mechanistic pathways directly depending on the temperature based on the scaling factor *n* calculated [36]. As a result, the better fit to a kinetic model for cyanide polymerization is directly dependent on the working temperature. However, none of the kinetic models or kinetic approaches mentioned above were suitable when NH_4_CN polymerization was assisted by MWR, for two main reasons: (i) the use of MWR renders it impossible to acquire conversion values for reaction times less than 2 min due to the necessary thermal heating ramp to reach the desired temperatures and, therefore, the reaction kinetics cannot be properly studied by thermogravimetric methods; and (ii) conversion values, chemical composition and parameters calculated from the Fourier-transform infrared (FTIR) spectra showed fluctuating and apparently random behavior along the reaction time [10,37], which was not previously observed in these systems when CTH was used [29,35,36]. In fact, the use of the MWR seems to make it more difficult to predict the evolution of the cyanide polymerization system over time and, consequently, a direct and guided modification of the desired properties of the reaction products is not obvious. Note that the characteristics of HCN-derived polymers can change notably along the reaction time but, in general, in an almost linear and easily interpretable way when CTH synthetic methods are used [29]. Therefore, taking into account the applicability of the statistical tools for the synthetic design and characterization and classification of polymeric systems [44,45,46], the main goals of this study are to help improve and develop synthetic methods for the generation of new soft materials based on HCN chemistry and to understand the plausible behavior of cyanide in prebiotic alkaline hydrothermal systems using multivariate analysis. In concrete terms, the progress of cyanide polymerizations assisted by MWR over time at three different temperatures was systematically evaluated using multivariate analysis. In the same way, cyanide polymerizations at 80 °C using CTH were used as control syntheses to determine the influence of the MWR in the generation of cyanide polymers. Principal component analysis for categorical data (CATPCA) is used mainly to compare the chemical evolution of a system over time regarding the temperature and the characteristics of the final reaction products. In addition, the chemometric processing of the FTIR and UV-Vis spectra and the thermogravimetry (TG), derivative thermogravimetry (DTG) and differential scanning calorimetry (DSC) curves have long been known in several analytical areas, such as food identification [47], crude oil analysis [48], fossil dating [49] and studies of woods [50]. Here, for the first time, principal component analysis (PCA) was performed with a dataset from FTIR and UV-Vis spectra and thermal analysis of NH_4_CN polymers. Chemometric analysis of the FTIR spectra and of the thermogravimetric analysis (TGA) and DTG curves was performed to properly interpret the properties of the gel fractions (insoluble cyanide polymers, since hydrothermal cyanide polymerization is a precipitating reaction). Moreover, a multivariate analysis of the UV-Vis spectra of the sol fractions (water-soluble oligomers/polymers) was carried out in an attempt to relate the progress of the gel fractions to their corresponding sol fractions.

The multivariate analytical results shown herein indicate that the production of cyanide polymers is very reproducible for fixed specific reaction conditions and that equivalent compositions and thermal final properties of the products can be reached when the reaction time is properly chosen, even when working at different synthetic temperatures. These aspects are discussed from the point of view of prebiotic chemistry and under the design of new soft materials.

## 2. Materials and Methods

### 2.1. Synthesis of the NH_4_CN Polymers

**Figure 1:**
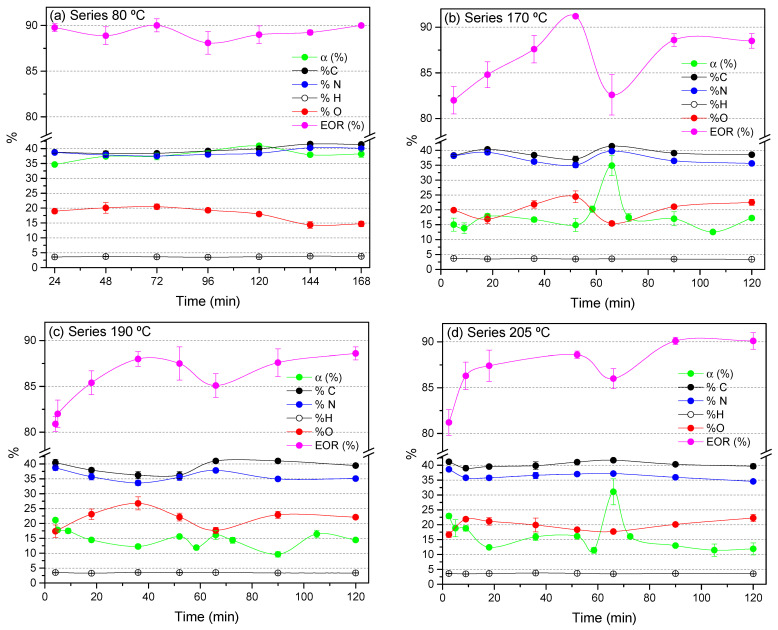
Evolution of NH_4_CN polymerization over the reaction time. (**a**) Series 80 °C is shown as representative control experiments for cyanide polymerization using CTH; and (**b**) series 170 °C, (**c**) series 190 °C and (**d**) series 205 °C of cyanide polymerizations assisted by MWR. Green lines = conversion degree, α (%), where α (%) = [(initial mg of CN^−^)/(final mg of insoluble polymer)] × 100 (for details, please see Table S1); black lines = % C; blue lines = % N; red lines = % O; pink lines = extension of the reaction, EOR (%), where EOR (%) = [I_1640_/(I_2200_ + I_1640_)] × 100, calculated from the intensity of the respective features of the FTIR spectra (for details of elemental analysis data and EOR values, please see Table S2). At least three independent experiments were considered for each point reported, i.e., at least three independent samples were measured. The average values together with their corresponding standard deviations are presented.

The NH_4_CN polymers synthesized in this work were obtained following the methodology detailed in [10,37] using a Biotage Initiator^+^ microwave reactor purchased from Biotage (Uppsala, Sweden). The initial equimolar concentrations of NaCN and NH_4_Cl were always 1 M. The synthesis of the control polymers prepared at 80 °C is described in [29]. All syntheses were carried out in triplicate. The details about the reaction time and temperature for each reaction and the conversion values reached for each case are described in Table S1. All the corresponding average values of conversion, α (%), are shown as green lines in Figure 1.

All security and safety measures were taken during the development of the experiments, considering the safety information provided by the Sigma-Aldrich supplier (St. Louis, MO, United States) about NaCN and NH_4_Cl. On the other hand, although HCN-derived polymers have been shown as biocompatible [19,20,21,22,23,24], the cyanide polymers synthesized in the present work were handled with the same security measures as NaCN.

### 2.2. Instrumental Analysis

All the parameters and equipment for the measurements of elemental analysis, FTIR spectra and thermal analysis are described in [29]. The UV-Vis spectra were recorded, and the raw data were properly handled, as defined in [36,51].

### 2.3. Statistical Analysis

Principal component analyses (PCAs) using the compositional and spectroscopic characteristics of the polymers as variables, such as α (%), % C, % H, % N, % O, C/O, N/O, C/N and C/H molar ratios and extension of reaction (EOR (%)), were carried out. The goal of principal component analysis is to reduce an original set of variables into a smaller set of uncorrelated components that represent most of the information found in the original variables. The technique is most useful when a large number of variables prohibits effective interpretation of the relationships between objects (subjects and units). By reducing the dimensionality, you interpret a few components rather than a large number of variables. Tests were performed using the multivariate data analysis software CANOCO 4.5 (Microcomputer Power, Ithaca, NY, USA) [52]. The program CANODRAW 4.0 (in the Canoco package) was used for graphical presentation.

Bivariate Pearson’s correlation coefficients were calculated to examine trends between physical categorical variables (temperature and time) and physicochemical characteristics detected in the different experiments. Transformation of data was not required to satisfy the assumption of normality. Principal component analyses (PCAs) of TGA and DTG curves and FTIR and UV-Vis spectra were performed. Furthermore, using the obtained component-scoring coefficients, hierarchical dendrograms based on the Euclidean squared distance method were calculated for each case. With physical categorical and physicochemical characteristics, categorical principal component analysis (CATPCA) was performed, and activity count cutoff points were estimated using bootstrapping methods. This procedure simultaneously quantifies categorical and numerical variables while reducing the dimensionality of the data. Standard principal components analysis assumes linear relationships between numeric variables. On the other hand, the optimal scaling approach allows variables to be scaled at different levels. Categorical variables are optimally quantified in the specified dimensionality. As a result, nonlinear relationships between variables can be modeled. The technique is most useful when numerous variables prohibit effective interpretation of the relationships between objects (subjects and units). Bivariate Pearson’s correlation coefficients, PCA, hierarchical dendrograms and CATPCA were calculated using IBM SPSS Statistics 27.

## 3. Results

### 3.1. CATPCA and PCA to Study Cyanide Polymerization Assisted by MWR Tracking by Means of Gel Fractions

#### 3.1.1. Conversion Limit, Chemical Composition and Data from FTIR Spectroscopy

Cyanide polymerization assisted by MWR is described using three different temperatures, 170, 190 and 205 °C. These temperatures were chosen on the basis of a previous report about microwave-driven cyanide polymerization [37]. In addition, syntheses at 80 °C using CTH have been considered representative control experiments to determine the effect of the MWR. Note that the conversion limit for these CTH polymerizations was claimed to be approximately 24 h at 80 °C [35,36]. Therefore, considering the manufacturer’s indications for the microwave reactor, as an example, 11 h at 80 °C is equivalent to 1 min at 170 °C, and all the polymerizations herein considered with reaction times from 2 min to 2 h would have reached their conversion limit. However, a clear fluctuating behavior is observed for the conversion values vs. reaction time when detailed representations are made (Figure 1, green lines). For the control polymerizations at 80 °C, conversions between 35 and 41% were reached using reaction times between 24 and 168 h [29] with an average value of 38 ± 2% (Figure 1a). Considering the value of this standard deviation, the relative fluctuation for the conversion is approximately 5% in the mentioned range of time considered (Figure 1a, green line). However, this fluctuation is more evident when the polymerizations are assisted by MWR. Thus, for 170 °C, 190 °C and 205 °C, the average conversions are 16 ± 2%, 15 ± 3% and 15 ± 4% (Figure 1b–d, green lines), respectively. The greatest oscillations were observed at 205 °C, with a relative standard deviation of approximately 27% with respect to the conversion average value. Note that the profile of the curve conversion vs. reaction time is different for each temperature. For example, for 205 °C, the conversion degree is higher for a reaction time of 2 min than for 2 h. In contrast, for 170 °C, these values are similar for 2 min or 2 h. Additionally, it is important to indicate that the points registered at 67 min for 170 °C and 205 °C were not considered for the calculations of these conversion averages due to the exceptional data reached, 35 ± 3% and 31 ± 4%, respectively. This exceptional point is not observed in the 190 °C series (Figure 1c). In any case, comparatively, for all the time ranges studied here, the MWR leads to lower yields for insoluble NH_4_CN polymers at any temperature than CTH, except for the two unexpected points indicated at 67 min. This general result is in agreement with the data previously reported, which indicated that an increase in the temperature leads to lower conversion values [35,36] and that the MWR improves the oxidation and hydrolysis processes of cyanide and the intermediate products during the polymerization reactions, decreasing the overall yield for the insoluble macromolecular fractions [30,37]. Therefore, an important effect of the MWR is to reduce the hydrothermal production of insoluble cyanide polymers. In general, this decrease in average values seems independent of the working temperature when MWR is used, considering the average conversions cited above for each thermal series considered herein.

**Figure 2:**
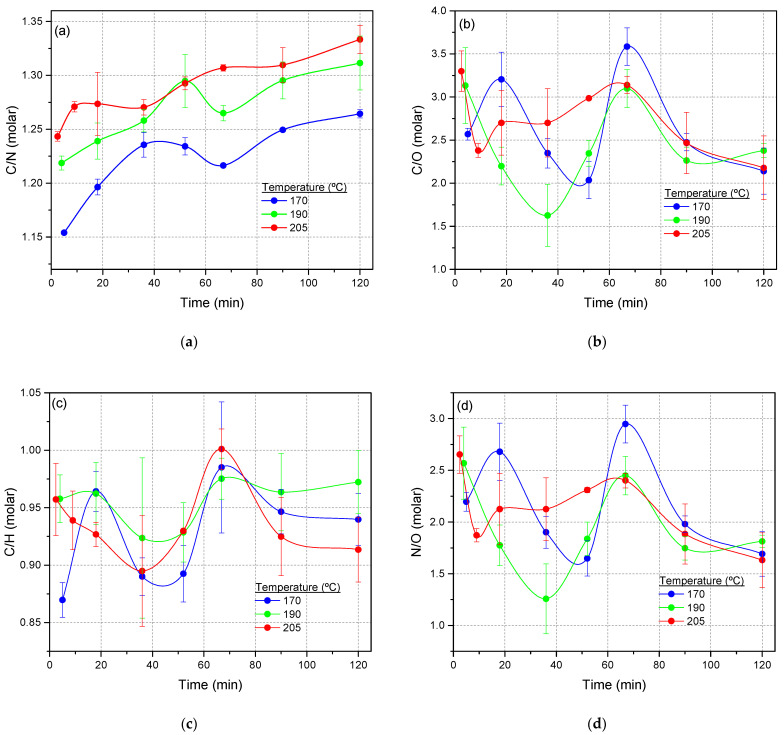
Evolution of the molar relationships for the three series of experiments carried out using MWR against reaction time (for details, please see Table S2). (**a**) C/N molar relationships; (**b**) C/O molar relationships; (**c**) C/H molar relationships and (**d**) N/O molar relationships. These molar relationships represent the average values of three independent samples for each reaction condition.

With respect to the evolution of the chemical compositions of the cyanide polymers, in the 80 °C series, we observed that the % C (black line) is slightly increasing and the % O is decreasing (red line) with the reaction time (Figure 1a). However, this clear and almost lineal behavior observed for the reactions at 80 °C is not all appreciated in the progress of the elemental composition of the polymers synthesized at higher temperatures (Figure 1b–d). However, a detailed representation of the C/N molar relationships (Figure 2a) strongly indicates that the longer reaction times and the higher temperature leads to the generation of macromolecular systems poorer in nitrogen than shorter reaction times at the lower temperature. Thus, the polymers produced at 205 °C are the poorest in nitrogen (Figure 2a). This fact might be related to deamination processes during cyanide polymerization, which might be favored at high temperature, leading to the generation of N-heterocycles, as proposed previously [6]. On the contrary, the curves showing C/O, C/H and N/O molar relationships vs. reaction time (Figure 2b–d, respectively) do not show a direct and easy association between the evolution of the chemical composition and the reaction time. Further, note that the C/O, C/H and N/O molar relationships present the highest values at the singular time of 67 min for the three series.

**Figure 3:**
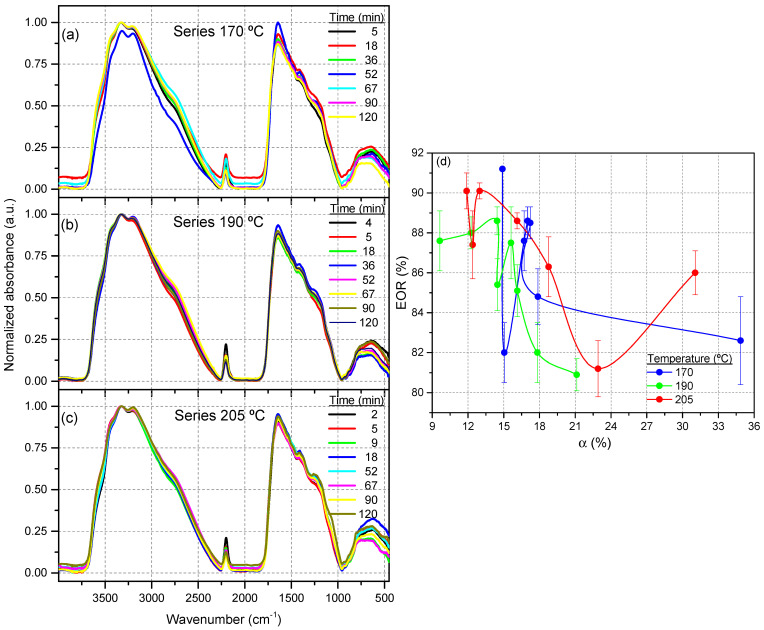
(**a**–**c**) Representative FT-IR spectra of NH_4_CN polymers synthesized at the indicated temperatures and using the shown reaction times in each graph, by assisted MWR to reach the corresponding temperature and from equimolar water solutions of NaCN and NH_4_Cl (1 M). (**d**) Values of EOR (%) vs. conversion degree for cyanide polymers synthesized at the temperatures indicated in the legend using MWR. The average EOR (%) values were calculated from the data shown in Table S2.

Similar results are observed when structural data are considered. The EOR (%) (Figure 1, pink lines) was calculated from the intensities of some FTIR spectroscopic features, as follows: EOR (%) = [I_1640_/(I_2200_ + I_1640_)] × 100, considering the study of conventional nitrile polymers [53]. The peak centered at ~1640 cm^−1^ can be assigned to conjugated C=C, to (C=N)_n_ structures or to a combination of both, and the band centered at ~2200 cm^−1^ is related to nitrile groups. Representative FTIR spectra of the insoluble NH_4_CN polymers obtained using MWR are shown in Figure 3. An exhaustive interpretation of these FTIR spectra has been made comprehensively elsewhere (see, e.g., Refs. [54,55,56,57,58]); therefore, an exhaustive interpretation is not considered herein. The value of the EOR (%) varies from 0 for no reaction to 100% for a complete conversion of the nitrile groups. The EOR (%) is well established to increase with the degree of conversion when CTH is used to produce cyanide polymers [35,36]. Thus, if the standard errors are considered, the EOR (%) values for the 80 °C series are practically constant along the timeline since the conversion limit has been reached (Figure 1a, pink line). The EORs have an average value of 89 ± 1% for the reaction times considered. In contrast, the EOR (%) values for the other three series of experiments apparently do not have direct and linear relationships with the degree of conversion (Figure 1b–d, pink lines). A detailed representation of the EOR (%) against conversion degree shows, in some cases, an opposite behavior, i.e., some points with the greatest values of conversion present the lowest EOR (%) data (Figure 3d). However, the EOR (%) seemed to increase with the reaction time, although in a nonlinear way, in the MWR experimental series (Figure 1). As in the case of the chemical composition commented above, the EOR (%) value presents a singular point at the time of 67 min for the three MWR series. 

**Figure 4:**
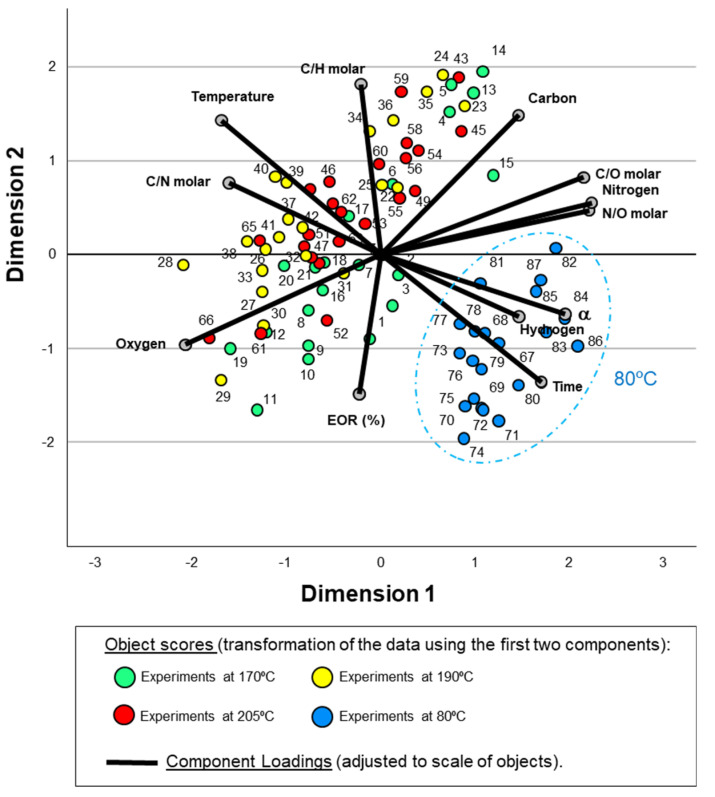
Categorical principal component analysis (CATPCA) biplot using as variables the experimental synthesis conditions used, reaction time and temperature, degree of conversion values, elemental composition data and EOR (%) from the FTIR spectra for the NH_4_CN−derived polymers obtained. A total of eighty seven independent experiments were considered in this study (Table S2). Component loadings of variables are indicated by vectors and object scores of experiments by dots. Green dots represent samples from the 170 °C series, yellow represents the 190 °C series, red represents the 205 °C series and blue represents the 80 °C series. The triplicates considered in this study were the reactions carried out using the experimental conditions detailed in Table S2. The number of experiments corresponds to the reactions in Table S1. The experiments are grouped into two clusters based on the variables: a heterogeneous group and a homogeneous group consisting of the experiments performed at 80 °C (surrounded by an ellipse).

At this point, statistical methods, as useful analytical tools, are used to provide a global and interpretable vision of the results discussed above because: (i) the relationships between the experimental synthetic conditions and the properties of the final products are difficult to find using qualitative analyses, and (ii) well-established kinetic models cannot be used to describe the NH_4_CN polymerization assisted by MWR, as indicated in the Introduction. Thus, CATPCA was performed to determine the statistical significance of all data and the robustness of the syntheses for the gel fractions described. For that determination, the triplicate data of a total of seven reactions from different reaction times for each series at 80 °C, 170 °C and 190 °C and eight polymerizations for the series at 205 °C were considered, i.e., the data from eighty-seven independent experiments shown in Figure 1 and Figure 2 (Table S2). The CATPCA analysis based on ten physicochemical characteristics (α (%), % C, % H, % N, % O, C/O, N/O, C/N and C/H molar ratios and EOR (%)) and two categorical variables (temperature and time) for 80 °C, 170 °C, 190 °C and 205 °C sorted the reactions under study into two defined groups. Thus, all reactions resulting at 80 °C grouped together (Figure 4). The first two dimensions of the CATPCA together explained 91% of the variance in the dataset and were supported by a total Cronbach’s α of 0.97 (maximum value = 1), indicating the high reliability of the detected pattern in the dataset [59]. The estimates and bootstrap 95% confidence intervals showed that variations in temperature, hydrogen and molar C/H were significantly higher than the rest, while the variations in nitrogen, oxygen, molar C/O and N/O were the lowest (Table S3, Figure S1). The EOR values present a positive correlation with the reaction time, with the % O and with C/N molar and, therefore, logically, a negative correlation with the % N. However, the EOR seems independent of the reaction temperature and shows an unexpected negative correlation with the conversion degree, α (%). The % O presents strong negative correlations with the % C and % N and a negative correlation with α (%) but, at the same time, shows a slight positive correlation with the reaction time and seems independent of the temperature. However, α (%) presents an unexpectedly low negative correlation with the reaction time and positive correlations with % C and % N, but it is independent of the temperature. In fact, the temperature only presents a positive correlation with the C/N molar. In this approach, including the 80 °C series data, a direct correlation between time and conversion is shown since the greatest conversion values are reaching for this series for the longest reaction times considered, but an inverse correlation between temperature and H% is observed, and a direct correlation between EOR and % O is conserved.

**Figure 5:**
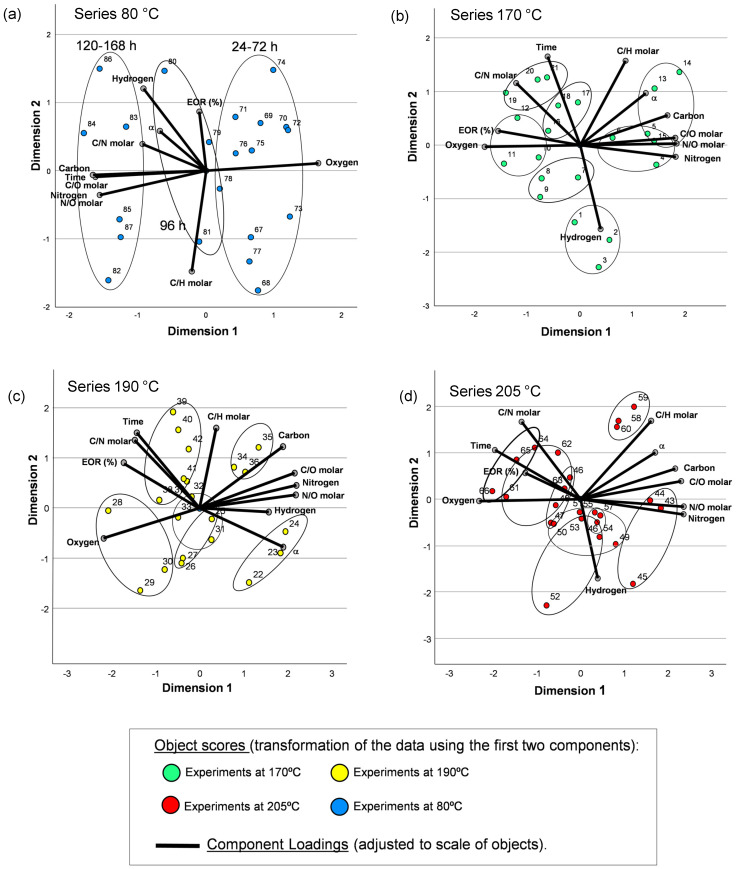
CATPCA biplot using the experimental synthesis conditions used, reaction time, conversion degree values, elemental composition and calculated EOR data (%) from the FTIR spectra for the NH_4_CN-derived polymers obtained as variables. Component loadings of variables are indicated by vectors and object scores of experiments by dots. In this analysis, each experimental series was analyzed independently: (**a**) 80 °C series; (**b**) 170 °C series; (**c**) 190 °C series; and (**d**) 205 °C series. The reaction time for each synthesis is indicated in Table S1. The ellipses surround the most related experimental results.

The greatest effect of the MWR seems to be to lead to lower conversion degrees independent of the reaction time and even of the temperature, in strong agreement with the above qualitative analysis of the data. Thus, the MWR influence on the conversion degree in the cyanide polymerizations beyond the working temperature and even the reaction time is statistically consistent. These lower conversion values, with respect to the procedures using CTH, could be related to decomposition, oxidation and hydrolysis secondary processes during the aqueous cyanide polymerization, as discussed in detail in [30,37]. Because the reaction temperature does not seem to have a highly relevant statistical influence on the chemical composition and some of the spectroscopic properties of the cyanide polymers synthesized using MWR, we decided not to consider this variable in our further statistical analysis. In this way, it was possible to more clearly see the effect of the reaction time on the chemical composition properties of our polymeric system and to determine which reaction conditions are more robust and favorable to obtain a cyanide polymer with a concrete composition. Thus, CATPCA was made for each of the series of experiments at 170 °C, 190 °C and 205 °C (Figure 5a–c). In addition, a CATPCA for the 80 °C series was also carried out with comparative purposes (Figure 5d). It is a first view that the data for the points corresponding to the triplicate experiments are more dispersed in the 80 °C series than in the MWR polymerization experiments. Moreover, considering these groupings, the experiments at 170 °C seem to be the more reproducible series. Interestingly, while for the series at 170 °C and 205 °C, the conversion has strong direct correlations with the % C and with the C/H molar being independent of the reaction time, for the 190 °C, the conversion values are strongly related to the % H and are inversely correlated with the reaction time. However, in all cases, including both MWR and CTH, α (%) presents an inverse correlation with % O, i.e., when a greater value of α (%) reaches a minor value of % O in the macrostructure of the NH_4_CN polymers. However, the % O is strongly directly related to the EOR (%) values in the three MWR series but independent of the 80 °C series. In fact, this last result was previously described for NH_4_CN polymerization under CTH, where the EOR values directly increased with the α (%) values [35] (please compare Figure 5 with Figure 13 from reference [35]). Thus, the EOR values obtained from cyanide polymers synthesized at 75–90 °C using CTH showed positive correlations with the C/N ratios but were independent of the % O and the C/O and N/O ratios [35]. In contrast, in the present case, for analogous cyanide polymerizations assisted by MWR, the EOR values present a strong direct correlation with the % O, strong negative correlations with the C/O and N/O ratios and a direct correlation with the C/N molar relationship. This result, considering the EOR values, will also be reflected in the PCA of the FTIR spectra of all these samples, as will be shown in the next sections.

Therefore, the cyanide polymerizations assisted by MWR seem to be more reproducible and robust than the cyanide polymerizations carried out using CTH, especially those performed at 170 °C, according to the grouping of the triplicate experiments (marked as ellipses in Figure 5). However, the prediction of the evolution of the chemical characteristics of these macromolecular systems over time is not obvious when MWR is considered for their syntheses. In contrast, the CTH experiments can be grouped following a timeline in short reaction times (24–72 h), intermediate reaction times (96 h) and long reaction times (120–168 h) with a clear statistical evolution of the system over time with respect to the chemical composition (Figure 5d).

As a result, the more relevant statistical conclusions of these first multivariate analyses are that increasing the reaction time leads to more reduced macrostructures with an increase in the conversion values when CTH is used, and on the contrary, MWR-assisted cyanide polymerizations produce more oxidized structures, especially at 205 °C, at longer reaction times, independent of the conversion values reached. Thus, the MWR is noteworthy to the cyanide polymerization processes beyond decreasing the conversion values, changing the elemental composition and the spectroscopic characteristics of the final products with respect to the analogous NH_4_CN polymers obtained using CTH. These spectroscopic differences have been appreciated by PCA for FTIR and UV-Vis spectra, as will be discussed in the next sections.

#### 3.1.2. PCA of the FTIR Spectra for the Cyanide Polymers

Generally, HCN-derived polymers show very similar FTIR spectra, independent of the experimental synthetic conditions used for their production, as shown in Figure 3 and widely shown in the literature [54,55,56,57,58,60]. However, the second derivative for the whole range of the IR spectrum, from 4000 to 400 cm^−1^, or for a specific spectral region is largely used together with chemometric methods for the classification and identification of samples with different natures [61,62,63]. Thus, as a first step to evaluate the suitability of the chemometric methods using the FTIR spectra for the classification of cyanide polymers, the 80 °C series was chosen. For PCA, using the whole spectra, different spectral regions and several Savitzky–Golay (SG) second derivatives with different window sizes of points were performed (Figures S2–S5).

**Figure 6:**
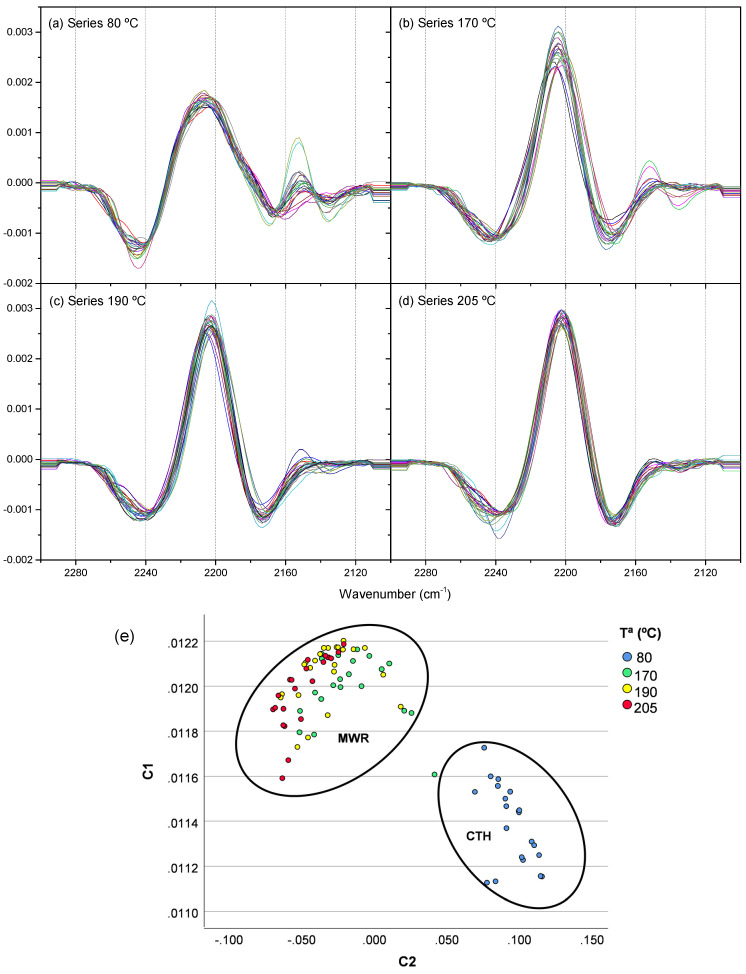
Second spectral derivatives in the FTIR nitrile spectral region (2300–2100 cm^−1^) from gel fractions of the cyanide polymerizations: (**a**) using conventional heating at 80 °C and (**b**–**d**) assisted by MWR at different temperatures, 170, 190 and 205 °C, respectively; (**e**) PCA of the dispersion of the second derivatives of the IR nitrile spectral region (2300–2100 cm^−1^). The FTIR spectra are from each of the triplicates of the samples synthesized using the conditions shown in Table S1 (please compare with the dendrogram in Figure S6). The second derivatives of the FTIR spectra of the 80 °C series correspond to the FTIR spectra previously reported in [29], shown here as control experiments. A total of ninety FTIR spectra were considered (Figure S6). Similar to Figure 4, the experiments are grouped into two main clusters: a heterogeneous group (MWR) and a homogeneous group consisting of the experiments performed at 80 °C (CTH).

The coefficient scores obtained in PCAs can be used to calculate hierarchical dendrograms. Dendrograms have the advantage that they allow one to see how experiments are grouped according to their spectral data, as shown in Figures S2–S5, where the clustering of the experiments based on the FTIR spectra can be seen (Figure S2); second derivative of the FTIR spectra (4000–450 cm^−1^) with 13 smooth points (Figure S3a) and 20 smooth points (Figure S3b); second derivative of the spectral IR region from 2300 to 2100 cm^−1^ with 13 points of smoothing (Figure S4a) or using 20 points of smoothing (Figure S4b); second derivative of the IR spectral region from 1820 to 950 cm^−1^ using 13 points of smoothing (Figure S5a) or with 20 points of smoothing (Figure S5b). As a better approach for a primary classification of the cyanide polymers, the second derivative of the 2300–2100 cm^−1^ spectral region with 20 smoothing points was selected, in good agreement with this region being considered the hallmark of HCN-derived polymers, as explained in detail in [35,58,60]. In addition, the EOR values are directly related to these features and, as discussed above, there is a different consistent statistical relationship between the EOR and the chemical composition of the polymers obtained by MWR or by using CTH. In the MWR-assisted polymerizations, the increase in the EOR values is directly related to a greater content of oxygen in the cyanide-derived macrostructures and, in contrast to using the CTH, the observed relationship is the opposite. Taking into account all these considerations, new statistical analyses were carried out using the second derivative of the nitrile spectral region (Figure 6), leading to classification into two main groups (Figure 6e). One group included all samples synthesized using CTH and the second group included those obtained using MWR. In addition, a certain subgrouping of the 170 °C and 205 °C series is also observed with the samples of the 190 °C series between the two other subgroups. This PCA explains 98% of the total variance with only two components. The same main classification can be observed better by the dendrogram shown in Figure S6. A very clear grouping of the samples from the 80 °C series is identified (Cluster I), the samples from the 170 °C series also present an ordered grouping (cluster III) with the exception of the triplicate experiments 13–15 (Table S1) (cluster II), which present a singular behavior, as mentioned above, with an exceptionally high value of conversion and a notably low EOR value (Figure 1b), and the experiments from the 205 °C series are clearly grouping (cluster II) with the exception of the triplicate 46–48 (Table S1). However, those samples prepared using equivalent reaction times based on the microwave reactor manufacture from the 190 °C and 205 °C series appear grouped together with one of the triplicates of the 170 °C series in cluster II, as was expected (samples 4, 22–24 and 43–45). Interestingly, the samples from the 190 °C series prepared using the longer reaction time are present in cluster II and those from the shorter reaction times are present in cluster III between the samples of the 170 °C series.

As a result, the PCA of the second derivative of the nitrile FTIR spectral region is in good agreement with the CATPCA grouping shown in Figure 4, providing, in this way, a practical classification of the cyanide polymers described herein. In addition, the PCA of the second derivative of the nitrile FTIR spectral region seems to be a suitable method for the first classification of cyanide polymers synthesized at different temperatures and secondarily with respect to the reaction time.

#### 3.1.3. PCA for the Thermal Analysis Data from the Gel Fractions

Thermal analysis techniques are considered useful tools for the distinction of HCN polymers with very similar FTIR spectra, and DTG and DSC are good fingerprints for the classification of these types of samples [64]. Moreover, the thermal analysis of HCN polymers synthesized under hydrothermal conditions has scarcely been explored [6,9,65]. Therefore, first, the TGA and DTG curves will be examined globally prior to the PCA study to obtain structural information, which could be related to the results discussed above.

**Figure 7:**
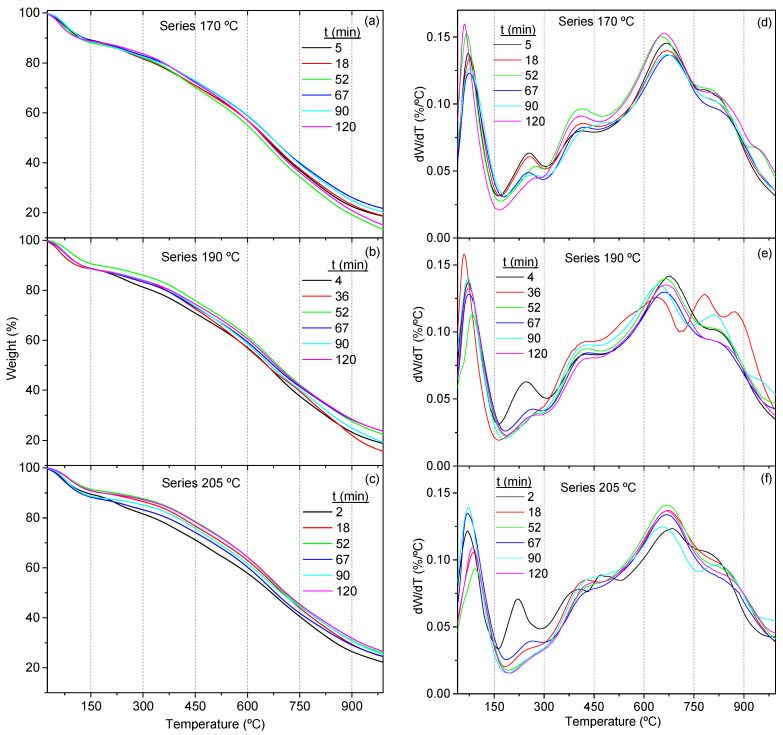
(**a**–**c**) Representative TG curves for NH_4_CN polymers synthesized at different temperatures and reaction times as indicated in the legends of the figure; (**d**–**f**) DTG curves corresponding to the TG curves.

**Figure 8:**
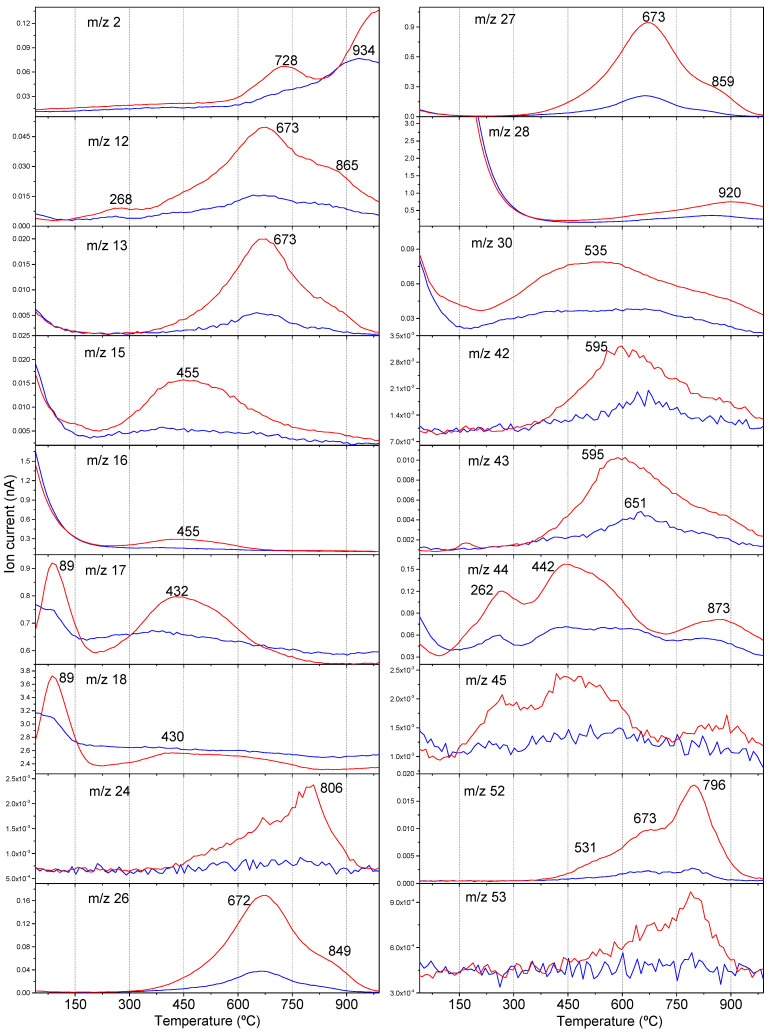
TG−MS curves for NH_4_CN polymers synthesized using MWR and water as solvent. Synthetic conditions of the NH_4_CN polymers, in blue: 170 °C/18 min; in red: 205 °C/2 h.

Significant differences were found through analysis of the thermal behavior of the different series from the MWR polymerizations (Figure 7). For example, the % in weight of the chars after heating the samples up to 1000 °C is higher for the series synthesized at 205 °C and notably greater than the percent of char in the CTH series (Figure 7a–c and Figure S7). Generally, the increase in the char for these types of polymers is related to a greater degree of cross-linking by oxidized groups, such as intermolecular amide bonds or intramolecular bonds, which lead to the formation of lactams, and by a greater conjugation in the system [64]. This fact can be related to higher oxidation in the macromolecular system that is observed for the series at 205 °C, but again, these relationships are not so clear for the series at 170 °C and 190 °C, unlike for the 80 °C series (please compare Figure 1 with Figure S7). In addition, annelation processes cannot be ruled out in the generation of the chars since the TG/MS curves show *m*/*z* 2 fragments linked to the loss of H_2_, which can be associated with the generation of new rings at high temperatures (Figure 8).

**Figure 9:**
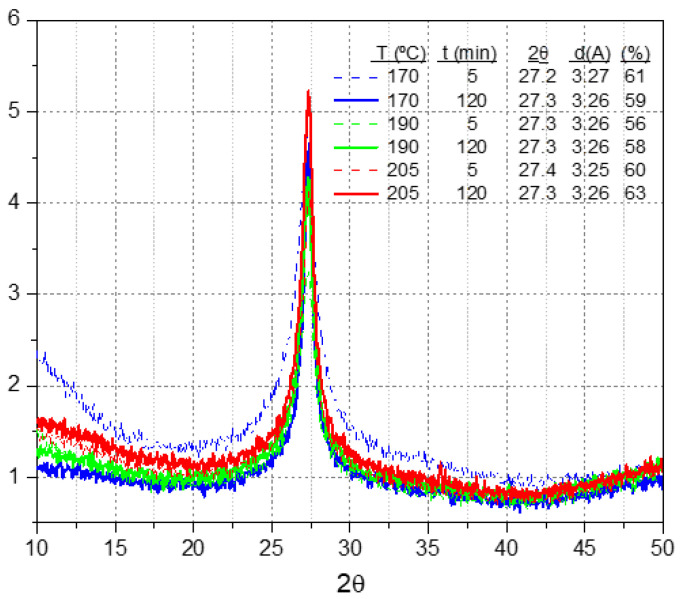
XRD patterns of NH_4_CN polymers synthesized at the temperatures and reaction times indicated, using MWR and water as solvent; d = interplanar distance; (%) = crystallinity of the sample.

The profiles of all the DTG curves shown in Figure 7d–f are similar to the profiles reported previously for other HCN-derived polymers and they will not be discussed in detail. However, the thermal peak observed at 220–260 °C assigned to the thermal decomposition of the weakest bonds [9] decreases with increasing polymerization temperature but also with increasing reaction time in the three MWR series. This last result was also observed for the 80 °C series (Figure 7d–f of this manuscript and Figure 6b of [29] for the 80 °C series). It seems that the longer reaction times and the increase in the synthesis temperature lead to the production of macrostructures with a minor presence of these thermal labile bonds. Based on the TG/MS curves, the weakest bonds are related to fragment *m*/*z* 44, which can be assigned to the loss of CO_2_ and/or HC(=NH)NH_2_ or HCONH-(Figure 8). This fragment was also observed in other HCN-derived polymers [64,65] but with different profiles. Considering the heterogeneous nature of the NH_4_CN polymers and the proposed hypothetical structures, it might be considered that the thermal decomposition step at ≈263 °C is related to decarboxylation processes of linear hydrolyzed polyamide structures (Scheme 4 in [6]) and the steps at 435 and 883 °C with thermal breakage of the unhydrolyzed polyamide structures (Scheme 4 in [6] and Scheme 1 in [64]). Therefore, at higher temperatures and longer reaction times, the presence of polyamide chains would be minor in the NH_4_CN polymers generated because the high synthesis temperatures for cyanide polymerization seem to increase the production of extended heterocyclic macrostructures. The other main TG/MS curves correspond to the fragments *m*/*z* 17 (NH_3_/-OH), 18 (NH_4_^+^/H_2_O), 26 (-CN) and 27 (HCN), which can be directly associated with the main loss weight steps shown in the DTG curves (please compare Figure 7d–f and Figure 8). Other TG/MS curves were observed: *m*/*z* 12 (C), *m*/*z* 13 (−CH−), *m*/*z* 15 (−NH−), *m*/*z* 16 (−NH_2_), *m*/*z* 24 (−C−C), *m*/*z* 28 (N_2_/CO), *m*/*z* 30 (NO), *m*/*z* 42 (−NCO), *m*/*z* 43 (HNCO/HOCN), *m*/*z* 45 (HCONH_2_), *m*/*z* 52 (−N=C-C=N−/−N=C−CH=CH) and *m*/*z* 53 (HN=C−CH=CH) (Figure 8). All these TG/MS peaks, herein indicated, were also reported previously for other HCN−derived polymers, and a more detailed analysis is beyond the scope of the present work. However, note that all these fragments observed in the samples from the MWR series were previously detected in HCN−derived polymers synthesized using CTH, although the profiles of the TG/MS curves were different. This result could indicate a similar but not identical structural characteristic, likely due to different proportions between the linear chains, the heterocycle chains, the extended macrocyclic structures and the presence of bidimensional macrostructures, as can be inferred from the DRX patterns of the samples from the cyanide polymerization assisted by MWR, Figure 9, and those previously reported for the 80 °C series and for the assisted MWR of cyanide polymerization at 180 °C (please see [10,37]).

**Figure 10:**
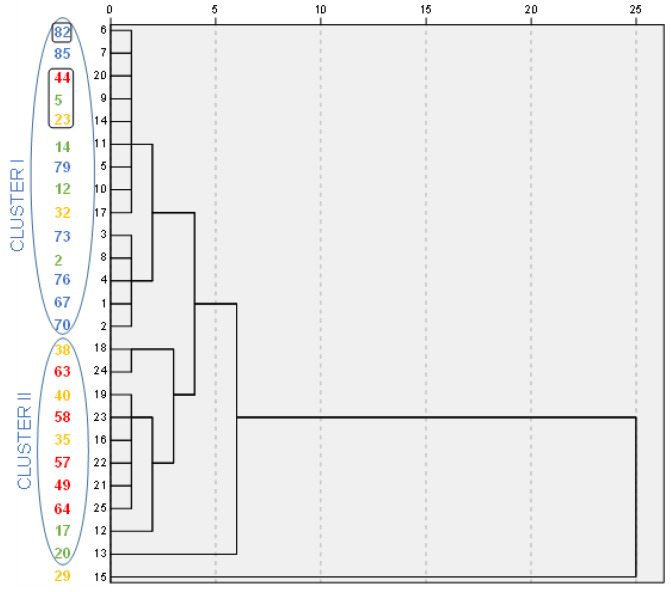
Hierarchical dendrogram based on PCA of DGT curves. Two clusters could be observed (indicated by ellipses). One of these groups (Cluster I) together, mostly samples taken at lower temperatures, and the other cluster (Cluster II) at higher temperatures or longer reaction times. The numeration of the experiments corresponds with Table S1. In blue, the samples from the 80 °C series, in green from the 170 °C series, in yellow from the 190 °C series and in red from 205 °C series. The samples in boxes are samples synthesized using equivalent reaction times based on the specifications of the microwave manufacturer.

To better interpret these thermal analytical results, an additional PCA of the DTG curves was carried out, and a suitable grouping was observed (Figure 10). This PCA explains 98% of the total variance with only two components. Again, the coefficient scores obtained in the PCAs were arranged in a hierarchical dendrogram where two clusters could be observed. The first cluster (Cluster I) shows a strong grouping of the four samples synthesized using equivalent reaction times; the rest of the samples from the 80 °C series and the samples from the 170 and 190 °C series were synthesized using shorter reaction times. In contrast, Cluster II shows the samples from the experiments carried out at the highest temperature, the 205 °C series, and the samples from the 170 °C and 190 °C series produced using the longest reaction times. In addition, only one sample is clearly ungrouped due to its particular DTG curve shape, as seen clearly in Figure 7e.

Thus, the PCA of the DTG curves indicates a clear clustering of the samples based on the temperature and the reaction time, in strong agreement with the results discussed above. Therefore, the shape of the DTG is statistically consistent for classifying HCN-derived polymers as a function of the synthetic conditions used for their production.

### 3.2. PCA for UV-Vis Spectra of the Sol Fractions

**Figure 11:**
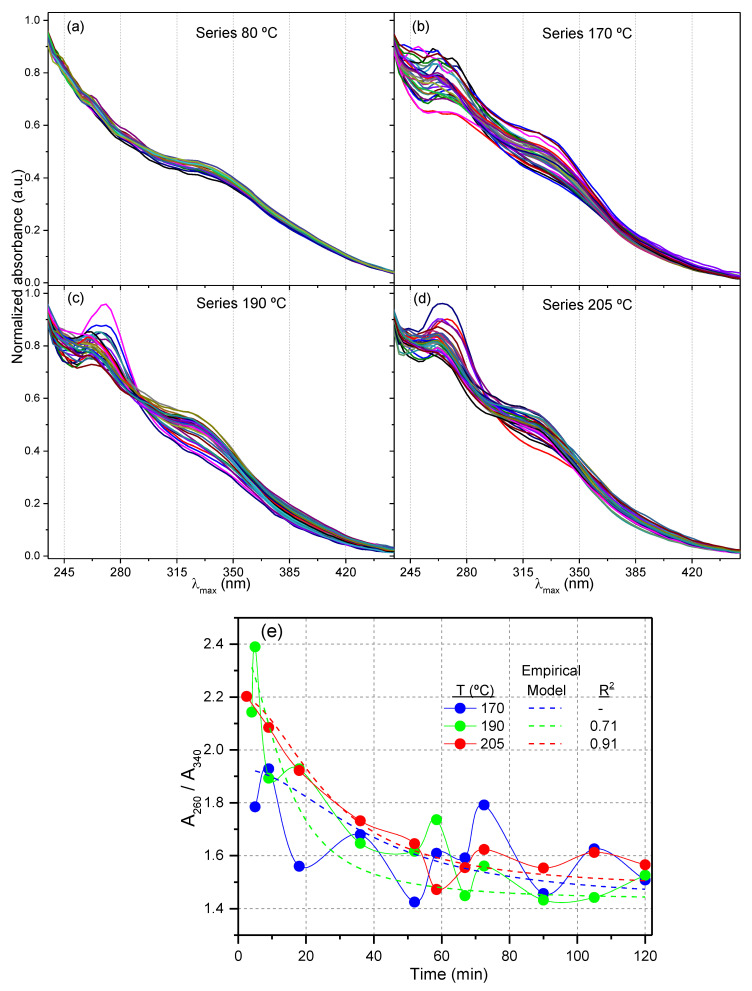
UV-Vis spectra of the raw soluble fractions (sol fractions) from the cyanide polymerizations: (**a**) using conventional heating at 80 °C, and (**b**–**d**) assisted by MWR at different temperatures, 170, 190 and 205 °C, respectively, from each of the triplicates of the samples synthesized using the conditions shown in Table S1; (**e**) evolution of the relationship between the intensity of the main bands observed in the UV-Vis spectra from the MWR series over time.

**Figure 12:**
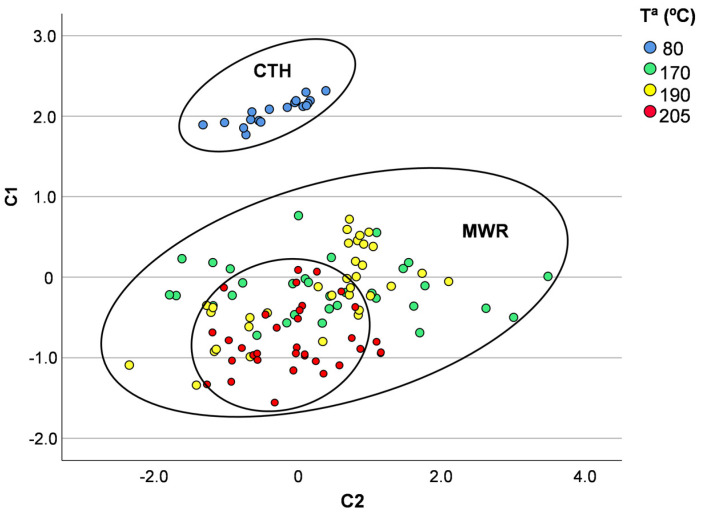
PCA of UV−Vis spectra data obtained (235–450 cm^−1^). Again, the experiments are grouped into two main clusters. A homogeneous cluster corresponding to the 80 °C series and a heterogeneous cluster with the rest of the series. In this analysis, the samples corresponding to the 205 °C series tend to appear together within the heterogeneous cluster. Ellipses mark these groupings.

The UV-Vis spectra of the soluble fractions from each reaction described above were registered to find complementary information about the progress of cyanide polymerization over time beyond the results obtained from the gel fractions and to compare the statistical results obtained from both fractions. Thus, the UV-Vis spectra of all soluble raw fractions (sol fractions) obtained at the same reaction times as their corresponding gel fractions (Table S1) were registered (Figure 11a–d). All the UV-Vis spectra from the MWR series show three main absorption bands centered at 230 nm, ~260 nm and ~340 nm (for a detailed assignment of these bands and their hypsochromic shifts, please see [10]). Herein, the bands at 230 nm are not considered for clarity in the figures and for a better statistical treatment of the data. Note that the intensity of the bands centered at ~260 and at ~340 nm change with the reaction time and that these bands are not observed in the UV-Vis spectra from the CTH series. Therefore, the relationships between the absorbance of these bands along the reaction time were represented to describe the progress of the cyanide polymerizations from the MWR series (Figure 11e), as was carried out in previous and directly related studies [10,51]. Specific details about the CTH series are discussed in [36] and, therefore, herein, they are not shown. A fluctuating behavior of the systems is observed at 170 °C and 190 °C, as expected based on previous results about the NH_4_CN polymerization assisted by MWR at 180 °C [10]. However, the fluctuation pattern is different for each temperature (Figure 11e). In contrast, the data from the 205 °C series can be fitted to a Hill equation that was made for syntheses at lower temperature using CTH [51]. Notably, the fitting shows a decreasing Hill curve and no growing function, as previously reported, indicating a significantly clear effect of the MWR in the cyanide polymerization process in the sol fractions. The approach to the timely progress to the polymerizations shown in Figure 11e indicates that the behavior of the system at 205 °C is different with respect to the other two MWR series and highly dissimilar to the CTH. In fact, the PCA of the UV-Vis spectra of all these samples indicates a clear grouping of the CTH and the MWR series with a reasonable subgrouping of the samples synthesized at 205 °C (Figure 12). This PCA explains 84% of the total variance by two components. The same results can also be observed from the dendrogram shown in Figure S8. A very strong grouping of the samples from the 80 °C series is identified in a clear cluster.

As a summary of all the results reported above, we can say that: (i) PCA studies from the FTIR spectra and the UV-Vis spectra show similar groupings, indicating a strong grouping of samples from CTH experiments due to a significant effect of the MWR; (ii) CATPCA using the data from chemical composition, conversion values and EOR (%) from the gel fractions provides very similar groupings of the samples to PCA studies using spectral data. In this way, all the data used in the present work statistically indicate a very robust and experimentally highly reproducible cyanide chemistry; (iii) despite the apparently random behavior of cyanide polymerizations assisted by MWR over time, the PCA of the FTIR and UV-Vis spectra points out the grouping of the samples as a function of the temperature, observing acceptable groupings of the samples from the series at 170 °C and 205 °C, with the samples at 190 °C inserted between them. Thus, although detailed kinetic studies were not possible, as in the case of the CTH reactions, again, the effect of the temperature is observed in the cyanide polymerizations assisted by MWR as well as in the thermal properties of the final obtained products.

## 4. Concluding Remarks and Outlooks

Hydrothermal cyanide polymerization has been statistically proven to be a very complex system.

The studies on NH_4_CN polymerization assisted by MWR prove, once again, that the cyanide polymerization processes depend on the temperature and that the properties of the reaction products are directly related to the temperature and the reaction time chosen for their synthesis. The evolution of the chemical composition of the cyanide polymers over time cannot be predicted when the polymerizations are assisted by MWR nor can the conversion limit values be predicted, as demonstrated for the cyanide polymerizations carried out using CTH. However, working at different temperatures, the election of equivalent reaction times leads to cyanide polymers with similar spectroscopic properties and very similar thermal stability. In addition, for fixed reaction conditions, the MWR ensures a stronger reproducibility for the cyanide polymer syntheses than the CTH, which was statistically confirmed.

Recently, analysis for N-heterocycles of the sol fractions from experiments simulating aerial and subaerial alkaline hydrothermal systems using CTH and MWR, respectively, has been shown to lead to the identification of mostly triazines in the first case and pyrimidines for the second one [11]. Considering these previous results and the statistical analysis discussed herein, if the MWR experiments are good simulations of subaerial alkaline hydrothermal systems, the cyanide chemistry in the subaerial part of the systems would be truly different from the cyanide chemistry in the aerial part. Assuming pressure gradients across the hydrothermal environments and considering that the pressure could play an important role in the diffusion processes during the cyanide polymerizations, this factor may be responsible for the significant differences observed between the CTH and MWR experiments. Kinetic studies in CTH cyanide polymerizations have shown that a first chemical control step is followed by diffusion control [36]. The working pressures of the MWR experiments of the 170 °C, 190 °C and 205 °C series were 10, 14 and 18 bars, respectively. They are the maximum pressure reached in each series of experiments and are directly dependent on the temperature chosen during the microwave-assisted polymerizations [10]. On the contrary, the 80 °C series was carried out at ambient pressure. Therefore, the effect of diffusion under each reaction condition could be very different. Moreover, hydrolysis processes during cyanide polymerization can be increased not only by high temperature [37] but also by high pressure, and together with a variation in the diffusion effects, could be responsible for the decrease in the conversion limit values in the NH_4_CN polymerizations assisted by MWR. Cyanide polymerization is well known to be a very complex chemical system [12,13,14,16,66], but herein, it was clearly supported by statistical methods that the complexity of the system is clearly increasing due to the effect of the MWR. Therefore, if the MWR is adequate to mimic subaerial hydrothermal systems, the plausible prebiotic chemistry in these environments would likely be more intricate than the plausible prebiotic chemistry in the aerial part of the system. Moreover, in the line of work considering HCN polymers as keystones to understand the increasing molecular complexity in abiotic scenarios, the results here show a clear example of an approach to the “messy chemistry” proposed by Mamajanov and co-workers as a way to address some questions about the origins of life [67]. In fact, from a prebiotic and astrobiological point of view, the multivariate methodology using the second derivative of a particular spectral region can be useful as a proper tool for the classification of complex prebiotic substances, such as HCN polymers, as well as *tholins* [68], since both present, in some cases, very similar FTIR spectra, although their composition and properties are different and depend on the synthetic conditions used for their production [60,69]. Thus, the use of the PCA of the FTIR spectra from synthetic samples and observational data could be of great interest in astrobiological studies about the complex atmospheric chemistry of Titan (the largest moon of Saturn) since the role of HCN in the generation of the orange haze of this satellite has been proposed to be very significant [70,71,72]. In addition, HCN polymers have been suggested as the oldest organic substances in the Solar System [73,74]. The multivariate analysis of the FTIR spectra of organic extracts present in carbonaceous chondrites with respect to synthetic samples could provide clues about their formation in outer space.

Finally, the MWR offers the possibility to reduce the noteworthy reaction times and to increase the reproducibility and robustness of the syntheses with respect to the CTH polymerizations. Both aspects are of high interest on the industrial scale due to the growing interest of HCN polymers in the materials science field. Therefore, the present study encourages finding new reaction conditions to obtain HCN polymers by assisted MWR synthesis, leading to greater yields with low cost and using green solvents, knowing that the size of the particles would be lower than the size of the particles obtained under CTH [30,37].

## Data Availability

Not applicable.

## Supplementary Materials

The following supporting information can be downloaded at: https://www.mdpi.com/article/10.3390/polym15020410/s1, Figure S1: Variance accounted for by each question; Figure S2: Hierarchical dendrogram based in principal component analysis of FT-IR sectors normalized (Series 80 °C); Figure S3: Hierarchical dendrogram based in principal component analysis of 2nd derivate FT-IR spectra (4000–450 cm^−1^) (Series 80 °C); Figure S4: Hierarchical dendrogram based in principal component analysis of 2nd derivate FT-IR spectral region from 2300 to 2100 cm^−1^ (Series 80 °C); Figure S5: Hierarchical dendrogram based in principal component analysis of 2nd derivate FT-IR spectral region from 1820 to 950 cm^−1^ (Series 80 °C); Figure S6: Hierarchical dendrogram based in principal component analysis of the 2nd derivate of the FTIR spectral region from 2300 to 2100 cm^−1^; Figure S7: Evolution of the char (%) after heating of the samples at 1000 °C as function of the reaction time; Figure S8: Hierarchical dendrogram based in principal component analysis of the UV-vis spectra. Table S1: Reaction conditions for the production of NH_4_CN polymers from equimolar solutions of NaCN and NH_4_Cl (1 M); Table S2: Elemental analysis data and the EOR (%) values for NH_4_CN polymers; Table S3: CATPCA correlation matrix and eigenvalues.
