# Supplementary material for: Multivariate Analysis Applied to Microwave-Driven Cyanide Polymerization: A Statistical View of a Complex System
Source: Polymers (Basel). 2023 Jan 12;15(2):410. doi: 10.3390/polym15020410 (PMC9866860; doi:10.3390/polym15020410)
Supplement: Supplementary file 1 [file polymers-15-00410-s001.zip › polymers-2087073-supplementary.pdf]

MULTIVARIATE ANALYSIS APPLIED TO THE MICROWAVE-DRIVEN CYANIDE POLYMERIZATION: A STATISTICAL VIEW OF A COMPLEX SYSTEM

Cristina Pérez-Fernández, Elena González-Toril, Eva Mateo-Martí, Marta Ruiz-Bermejo

SUPPLEMENTARY INFORMATION

Table S1. Reaction conditions for the production of NH_4CN polymers from equimolar solutions of NaCN and NH_4Cl (1 M) assisted by microwave radiation (MWR), series at 170 ºC (green), 190 ºC (yellow) and 205 ºC (red) , or using conventional thermal heating, series 80 ºC (blue). The series at 80 ºC was taken as reference from [Ruiz-Bermejo et al. 2019]. All the converstion values, a (%), are represented in the **Figure 1** of the main text (green lines). Note that the syntheses were carried out by triplicate.

Experiment	T (ºC)	t (min)	α (%)	Experiment	T (ºC)	t (min)	α (%)	Experiment	T (ºC)	t (min)	α (%)	Experiment	T (ºC)	t (min)	α (%)
**1**	170	5	13	**22**	190	4	21	**43**	205	2	24	**67**	80	1440	35
**2**	170	5	17	**23**	190	4	22	**44**	205	2	23	**68**	80	1440	35
**3**	170	5	14	**24**	190	4	21	**45**	205	2	22	**69**	80	1440	34
**121**	170	9	12	**88**	190	5	17	**97**	205	5	16	**70**	80	2880	38
**122**	170	9	15	**89**	190	5	18	**98**	205	5	21	**71**	80	2880	41
**123**	170	9	15	**90**	190	5	18	**99**	205	5	20	**72**	80	2880	37
**4**	170	18	18	**118**	190	9	18	**46**	205	9	18	**73**	80	4320	37
**5**	170	18	19	**119**	190	9	17	**47**	205	9	20	**74**	80	4320	38
**6**	170	18	17	**120**	190	9	17	**48**	205	9	19	**75**	80	4320	40
**7**	170	36	16	**25**	190	18	15	**49**	205	18	12	**76**	80	5400	41
**8**	170	36	10	**26**	190	18	14	**50**	205	18	12	**77**	80	5400	39
**9**	170	36	7	**27**	190	18	14	**51**	205	18	13	**78**	80	5400	39
**10**	170	52	14	**28**	190	36	12	**52**	205	36	15	**79**	80	7200	39
**11**	170	52	14	**29**	190	36	13	**53**	205	36	16	**80**	80	7200	41
**12**	170	52	17	**30**	190	36	12	**54**	205	36	17	**81**	80	7200	40
**94**	170	58	20	**31**	190	52	16	**55**	205	52	16	**82**	80	8640	38
**95**	170	58	21	**32**	190	52	16	**56**	205	52	16	**83**	80	8640	39
**96**	170	58	19	**33**	190	52	15	**57**	205	52	17	**84**	80	8640	38
**13**	170	67	34	**124**	190	58	17	**91**	205	58	13	**85**	80	10080	40
**14**	170	67	39	**125**	190	58	17	**92**	205	58	11	**86**	80	10080	44
**15**	170	67	38	**126**	190	58	14	**93**	205	58	11	**87**	80	10080	38
**106**	170	72	16	**34**	190	67	12	**58**	205	67	36				
**107**	170	72	19	**35**	190	67	12	**59**	205	67	32				
**108**	170	72	17	**36**	190	67	12	**60**	205	67	36				
**16**	170	90	14	**115**	190	72	15	**100**	205	72	16				
**17**	170	90	19	**116**	190	72	14	**101**	205	72	15				
**18**	170	90	18	**117**	190	72	14	**102**	205	72	17				
**109**	170	105	13	**37**	190	90	10	**61**	205	90	13				
**110**	170	105	13	**38**	190	90	10	**62**	205	90	13				
**111**	170	105	12	**39**	190	90	9	**63**	205	90	13				
**19**	170	120	17	**112**	190	105	17	**103**	205	105	11				
**20**	170	120	18	**113**	190	105	15	**104**	205	105	14				
**21**	170	120	17	**114**	190	105	17	**105**	205	105	10				
				**40**	190	120	15	**64**	205	120	14				
				**41**	190	120	14	**65**	205	120	11				
				**42**	190	120	14	**66**	205	120	11				

Table S2. Elemental analysis data and the EOR (%) values for NH_4CN polymers synthezed using the indicated conditions of temperature and reaction time from equimolar solutions of NaCN and NH_4Cl (1 M) assisted by microwave radiation (MWR), series at 170 ºC (green), 190 ºC (yellow) and 205 ºC (red) , or using conventional thermal heating, series 80 ºC (blue).

Experiment	T (ºC)	t (min)	% C	% H	% N	% O	C/N molar	C/O molar	C/H molar	N/O molar	EOR (%)
**1**	170	5	38.64	3.68	37.45	20.24	1.20	2.55	0.88	2.11	90.23
**2**	170	5	38.32	3.63	38.74	19.31	1.15	2.65	0.88	2.29	83.10
**3**	170	5	37.90	3.71	38.31	20.09	1.15	2.52	0.85	2.18	80.95
**4**	170	18	40.81	3.58	39.89	15.73	1.19	3.46	0.95	2.90	75.86
**5**	170	18	40.52	3.43	39.69	16.36	1.19	3.30	0.98	2.77	83.83
**6**	170	18	39.64	3.44	38.39	18.53	1.20	2.85	0.96	2.37	85.75
**7**	170	36	38.95	3.58	37.10	20.38	1.22	2.55	0.91	2.08	85.95
**8**	170	36	38.22	3.58	35.74	22.47	1.25	2.27	0.89	1.82	87.92
**9**	170	36	37.90	3.61	35.83	22.66	1.23	2.23	0.87	1.81	88.80
**10**	170	52	37.97	3.57	35.92	22.55	1.23	2.25	0.89	1.82	91.31
**11**	170	52	35.95	3.44	34.20	26.42	1.23	1.81	0.87	1.48	90.89
**12**	170	52	37.29	3.38	35.01	24.32	1.24	2.04	0.92	1.64	91.26
**13**	170	67	41.08	3.45	39.37	16.11	1.22	3.40	0.99	2.79	80.18
**14**	170	67	41.86	3.36	40.20	14.59	1.21	3.83	1.04	3.15	83.19
**15**	170	67	41.21	3.71	39.50	15.58	1.22	3.53	0.92	2.90	84.49
**16**	170	90	38.77	3.49	36.16	21.58	1.25	2.40	0.93	1.92	88.63
**17**	170	90	39.44	3.41	36.82	20.34	1.25	2.59	0.96	2.07	87.87
**18**	170	90	38.93	3.42	36.40	21.26	1.25	2.44	0.95	1.96	89.34
**19**	170	120	36.60	3.34	33.71	26.36	1.27	1.85	0.91	1.46	87.62
**20**	170	120	38.20	3.36	35.19	23.26	1.27	2.19	0.95	1.73	89.19
**21**	170	120	38.85	3.39	35.98	21.79	1.26	2.38	0.96	1.89	88.69
**22**	190	4	39.16	3.40	37.68	19.76	1.21	2.64	0.96	2.18	77.79
**23**	190	4	40.72	3.63	39.00	16.66	1.22	3.26	0.94	2.68	81.42
**24**	190	4	41.35	3.53	39.36	15.77	1.23	3.50	0.98	2.85	80.29
**25**	190	18	38.76	3.27	36.90	21.09	1.23	2.45	0.99	2.00	84.28
**26**	190	18	37.42	3.24	35.39	23.95	1.23	2.08	0.96	1.69	85.16
**27**	190	18	37.54	3.35	34.82	24.31	1.26	2.06	0.94	1.64	86.79
**28**	190	36	33.52	2.78	27.88	35.82	1.40	1.25	1.00	0.89	88.11
**29**	190	36	35.37	3.32	33.00	28.32	1.25	1.67	0.89	1.33	88.67
**30**	190	36	37.10	3.52	34.20	25.18	1.27	1.96	0.88	1.55	87.12
**31**	190	52	38.88	3.60	36.42	21.11	1.25	2.46	0.90	1.97	87.15
**32**	190	52	39.13	3.43	35.74	21.70	1.28	2.40	0.95	1.88	88.78
**33**	190	52	38.59	3.45	34.31	23.65	1.31	2.18	0.93	1.66	86.25
**34**	190	67	40.62	3.48	37.45	18.46	1.27	2.93	0.97	2.32	86.01
**35**	190	67	41.50	3.48	38.50	16.53	1.26	3.35	0.99	2.66	85.78
**36**	190	67	40.87	3.56	37.49	18.10	1.27	3.01	0.96	2.37	83.62
**37**	190	90	38.77	3.42	35.39	22.43	1.28	2.30	0.95	1.80	85.95
**38**	190	90	38.08	3.37	34.30	24.25	1.30	2.09	0.94	1.62	87.92
**39**	190	90	39.53	3.29	35.15	22.03	1.31	2.39	1.00	1.82	88.80
**40**	190	120	39.89	3.32	34.73	22.06	1.34	2.41	1.00	1.80	88.63
**41**	190	120	38.91	3.42	34.97	22.70	1.30	2.29	0.95	1.76	87.82
**42**	190	120	39.47	3.40	35.53	21.60	1.30	2.44	0.97	1.88	89.29
**43**	205	2	41.62	3.58	38.96	15.86	1.25	3.50	0.97	2.81	82.64
**44**	205	2	41.34	3.52	38.73	16.42	1.25	3.36	0.98	2.70	80.16
**45**	205	2	40.47	3.66	38.13	17.75	1.24	3.04	0.92	2.45	80.73
**46**	205	9	39.31	3.38	36.07	21.23	1.27	2.47	0.97	1.94	84.82
**47**	205	9	38.74	3.50	35.44	22.33	1.28	2.31	0.92	1.81	86.29
**48**	205	9	38.79	3.49	35.75	21.96	1.27	2.36	0.93	1.86	87.85
**49**	205	18	40.67	3.63	38.25	17.46	1.24	3.11	0.93	2.50	88.64
**50**	205	18	39.13	3.57	35.24	22.07	1.30	2.36	0.91	1.82	87.29
**51**	205	18	39.93	3.58	36.26	20.24	1.28	2.63	0.93	2.05	86.23
**52**	205	36	38.39	3.81	35.47	22.33	1.26	2.29	0.84	1.82	86.48
**53**	205	36	40.11	3.61	36.66	19.63	1.28	2.73	0.93	2.13	88.46
**54**	205	36	40.98	3.72	37.58	17.73	1.27	3.08	0.92	2.42	87.91
**55**	205	52	41.03	3.69	36.86	18.43	1.30	2.97	0.93	2.29	89.04
**56**	205	52	41.00	3.67	37.16	18.18	1.29	3.01	0.93	2.34	88.36
**57**	205	52	40.98	3.67	37.01	18.34	1.29	2.98	0.93	2.31	88.41
**58**	205	67	41.37	3.49	37.02	18.12	1.30	3.04	0.99	2.34	86.82
**59**	205	67	41.94	3.42	37.38	17.25	1.31	3.24	1.02	2.48	85.26
**60**	205	67	41.64	3.49	37.13	17.74	1.31	3.13	0.99	2.39	88.32
**61**	205	90	38.12	3.57	33.58	24.74	1.32	2.05	0.89	1.55	90.42
**62**	205	90	40.34	3.51	35.88	20.27	1.31	2.65	0.96	2.02	89.62
**63**	205	90	40.19	3.61	35.92	19.92	1.31	2.69	0.93	2.06	90.23
**64**	205	120	40.21	3.60	34.80	21.40	1.35	2.51	0.93	1.86	90.74
**65**	205	120	39.04	3.51	34.32	23.13	1.33	2.25	0.93	1.70	89.53
**66**	205	120'	36.69	3.47	32.31	27.53	1.32	1.78	0.88	1.34	84.78
**67**	80	1440	39.07	3.54	38.98	18.42	1.17	3.06	0.92	2.62	90.08108
**68**	80	1440	38.76	3.44	38.73	19.08	1.17	3.04	0.94	2.60	87.73953
**69**	80	1440	38.56	3.73	38.31	19.40	1.17	3.02	0.86	2.57	89.45956
**70**	80	2880	38.05	3.62	37.46	20.87	1.19	2.98	0.88	2.52	89.57363
**71**	80	2880	38.91	3.77	38.54	18.79	1.18	3.05	0.86	2.59	88.1912
**72**	80	2880	38.09	3.75	37.56	20.61	1.18	2.99	0.85	2.52	87.57391
**73**	80	4320	38.32	3.53	37.47	20.69	1.19	3.00	0.90	2.52	85.68319
**74**	80	4320	38.05	3.76	36.99	21.21	1.20	2.98	0.84	2.48	89.50949
**75**	80	4320	38.88	3.55	38.10	19.48	1.19	3.05	0.91	2.56	90.51412
**76**	80	5400	39.22	3.53	38.00	19.26	1.20	3.07	0.93	2.55	91.18264
**77**	80	5400	38.80	3.42	37.87	19.92	1.20	3.04	0.95	2.54	87.2176
**78**	80	5400	39.58	3.56	38.24	18.63	1.21	3.10	0.93	2.57	88.95688
**79**	80	7200	39.75	3.63	38.22	18.41	1.21	3.12	0.91	2.57	89.92806
**80**	80	7200	40.24	3.84	38.70	17.23	1.21	3.15	0.87	2.60	90.28931
**81**	80	7200	39.88	3.43	38.42	18.28	1.21	3.13	0.97	2.58	89.70192
**82**	80	8640	41.61	3.62	40.70	14.08	1.19	3.26	0.96	2.73	87.34969
**83**	80	8640	41.07	3.90	39.59	15.45	1.21	3.22	0.88	2.66	89.05151
**84**	80	8640	41.90	4.01	40.54	13.57	1.21	3.28	0.87	2.72	89.42187
**85**	80	10080	41.47	3.64	40.26	14.65	1.20	3.25	0.95	2.70	89.95808
**86**	80	10080	41.67	4.04	40.41	13.89	1.20	3.27	0.86	2.72	90.04966
**87**	80	10080	41.03	3.73	39.70	15.55	1.21	3.22	0.92	2.67	85.52521

Table S3. CATPCA correlation matrix and eigenvalues (Figure 4). High positive values between two variables indicate that they are directly proportional. High negative values indicate that they are inversely proportional.

	Correlations Original Variables											
	T (ºC)	t (min)	α (%)	Carbon	Hydrogen	Nitrogen	Oxygen	C/N molar	C/O molar	C/H molar	N/O molar	EOR (%)
T (ºC)	1.000	-0.066	0.015	0.303	0.211	-0.180	-0.022	0.617	0.074	0.130	-0.016	0.052
t (min)	-0.066	1.000	-0.186	-0.048	-0.300	-0.424	0.294	0.561	-0.289	0.107	-0.324	0.503
	0.015	-0.186	1.000	0.564	0.093	0.588	-0.659	-0.244	0.669	0.438	0.675	-0.456
Carbon	0.303	-0.048	0.564	1.000	0.426	0.731	-0.864	0.007	0.880	0.521	0.844	-0.319
Hydrogen	0.211	-0.300	0.093	0.426	1.000	0.424	-0.399	-0.213	0.446	-0.475	0.414	-0.103
Nitrogen	-0.180	-0.424	0.588	0.731	0.424	1.000	-0.927	-0.599	0.896	0.339	0.929	-0.567
Oxygen	-0.022	0.294	-0.659	-0.864	-0.399	-0.927	1.000	0.395	-0.945	-0.478	-0.960	0.522
C/N molar	0.617	0.561	-0.244	0.007	-0.213	-0.599	0.395	1.000	-0.335	0.183	-0.444	0.526
C/O molar	0.074	-0.289	0.669	0.880	0.446	0.896	-0.945	-0.335	1.000	0.436	0.964	-0.520
C/H molar	0.130	0.107	0.438	0.521	-0.475	0.339	-0.478	0.183	0.436	1.000	0.437	-0.263
N/O molar	-0.016	-0.324	0.675	0.844	0.414	0.929	-0.960	-0.444	0.964	0.437	1.000	-0.565
EOR (%)	0.052	0.503	-0.456	-0.319	-0.103	-0.567	0.522	0.526	-0.520	-0.263	-0.565	1.000
Dimension	1	2	3	4	5	6	7	8	9	10	11	12
Eigenvalue	6.110	2.146	1.565	1.007	0.476	0.386	0.103	0.065	0.047	0.039	0.033	0.024

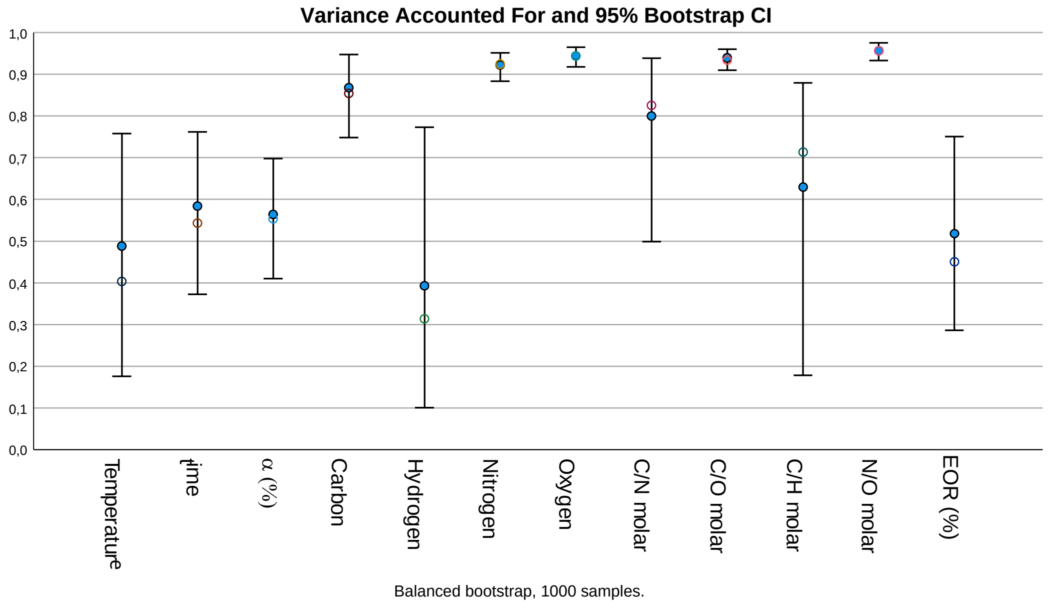

Figure S1. Variance accounted for by each question. Estimates and bootstrap 95% confidence intervals (CI) of variance on two categorical variables (temperature and time) and each spectroscopy characteristic. Temperature, hydrogen and molar C/H variations were significantly higher than the rest, while nitrogen, oxygen, molar C/O and N/O were the lowest.

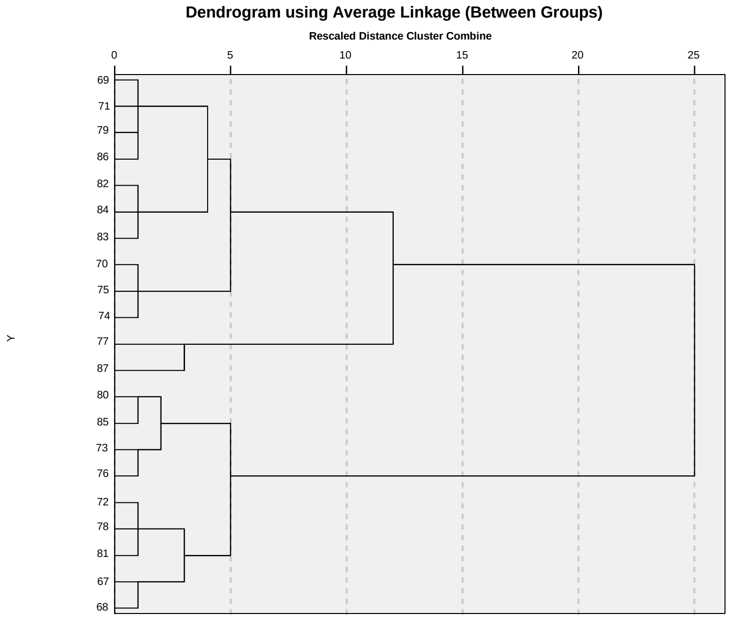

Figure S2. Hierarchical dendrogram based in principal component analysis of FT-IR sectors normalized (Series 80 ºC). To clarify PCA results (3.1.2.), coefficient scores obtained by PCA analyses were arranged in a hierarchical dendrogram. The numeration of the experiments correspond with the Table S1.

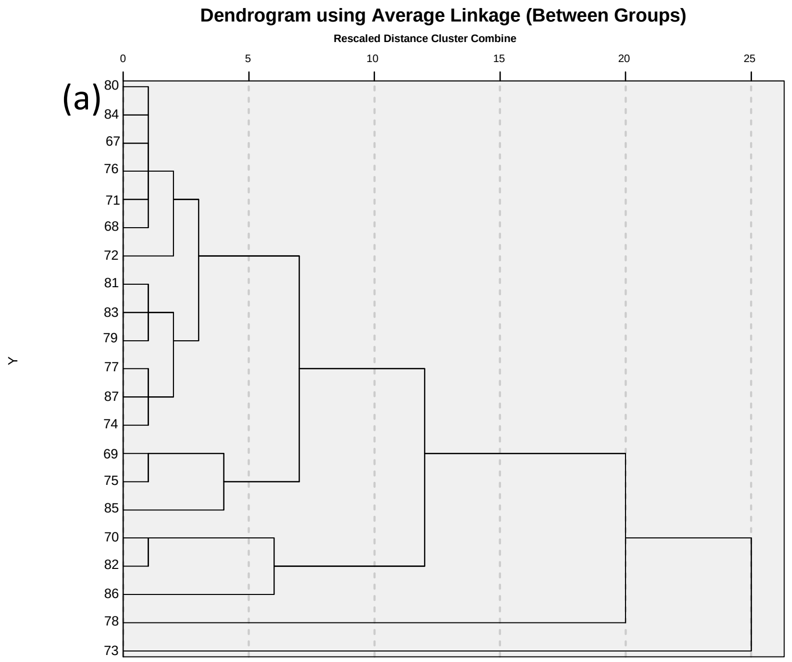

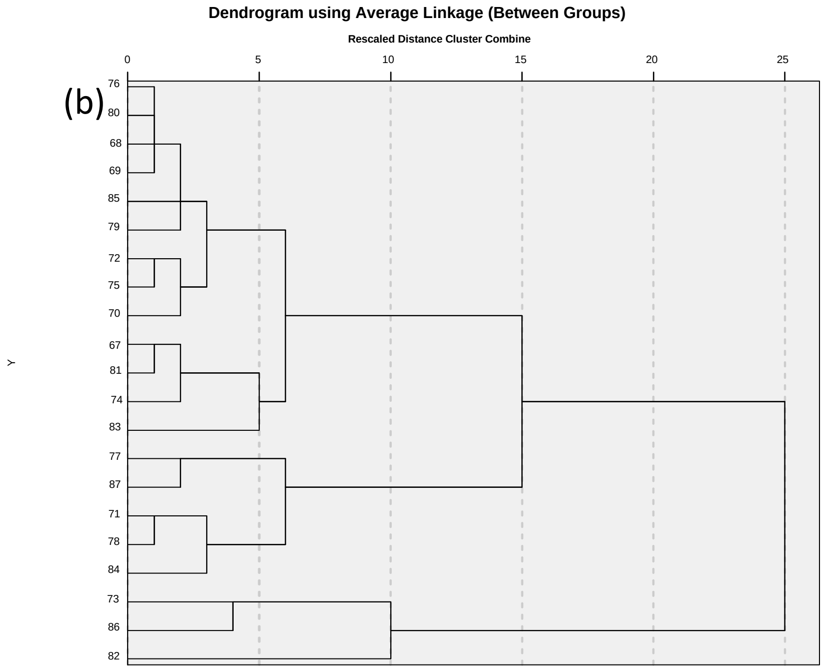

Figure S3. Hierarchical dendrogram based in principal component analysis of 2nd derivate FT-IR spectra (4000-450 cm^{-1}) (Series 80 ºC). To clarify PCA results (3.1.2.), coefficient scores obtained by PCA analyses were arranged in a hierarchical dendrogram. (**a**) considering 13 smooth points. (**b**) considering 20 smooth points. The numeration of the experiments correspond with the Table S1.

(a)

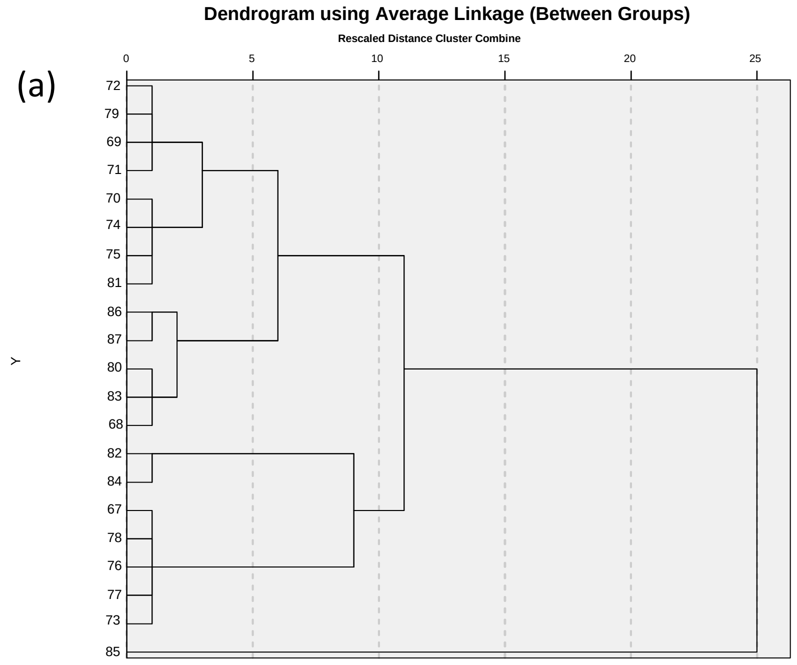

(b)

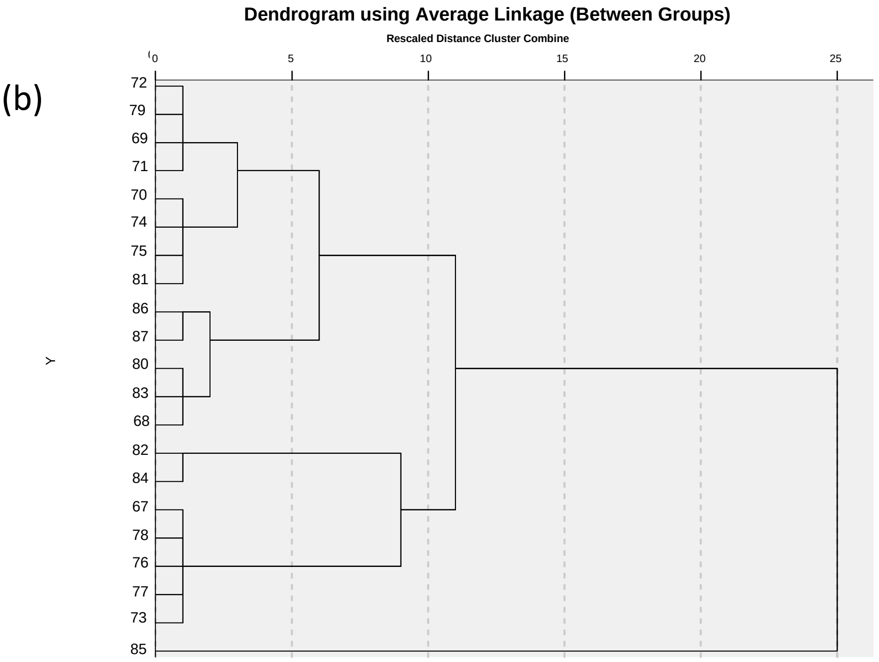

Figure S4. Hierarchical dendrogram based in principal component analysis of 2nd derivate FT-IR spectral region from 2300 to 2100 cm^{-1} (Series 80 ºC). To clarify PCA results (3.1.2.), coefficient scores obtained by PCA analyses were arranged in a hierarchical dendrogram. (**a**) considering 13 smooth points. (**b**) considering 20 smooth points. The numeration of the experiments correspond with the Table S1.

(a)

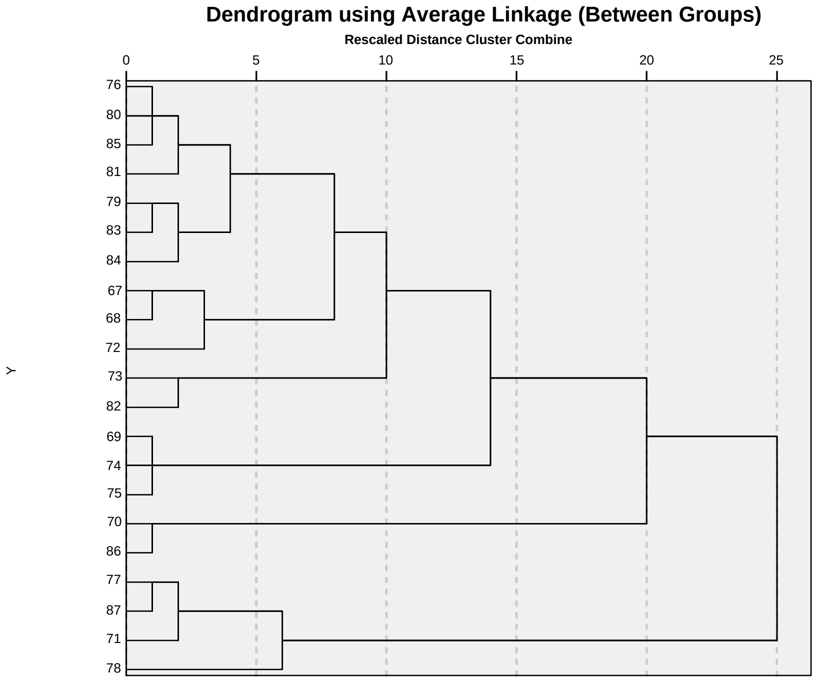

(b)

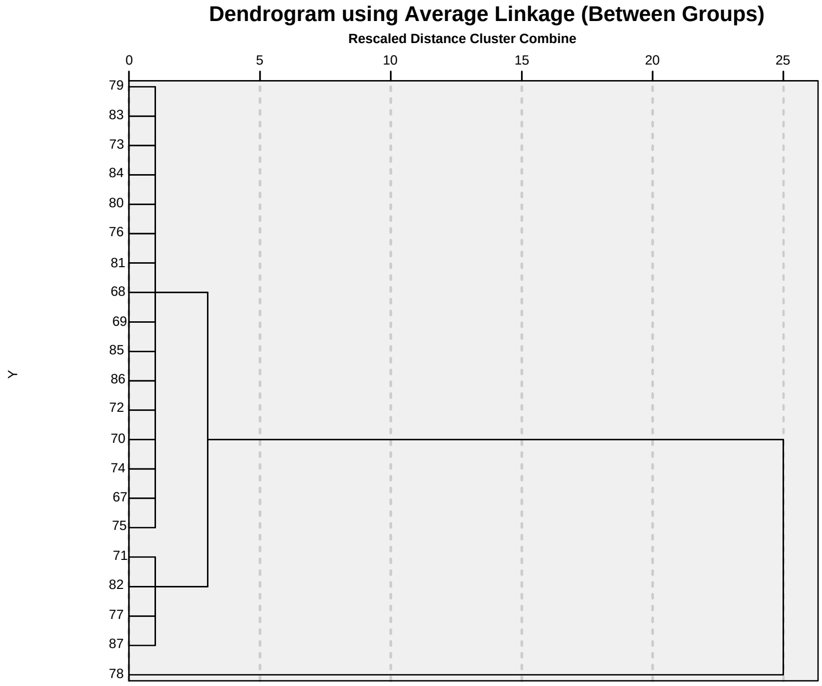

Figure S5. Hierarchical dendrogram based in principal component analysis of 2nd derivate FT-IR spectral región from 1820 to 950 cm^{-1} (Series 80 ºC). To clarify PCA results (3.1.2.), coefficient scores obtained by PCA analyses were arranged in a hierarchical dendrogram. (**a**) considering 13 smooth points. (**b**) considering 20 smooth points. The numeration of the experiments correspond with the Table S1.

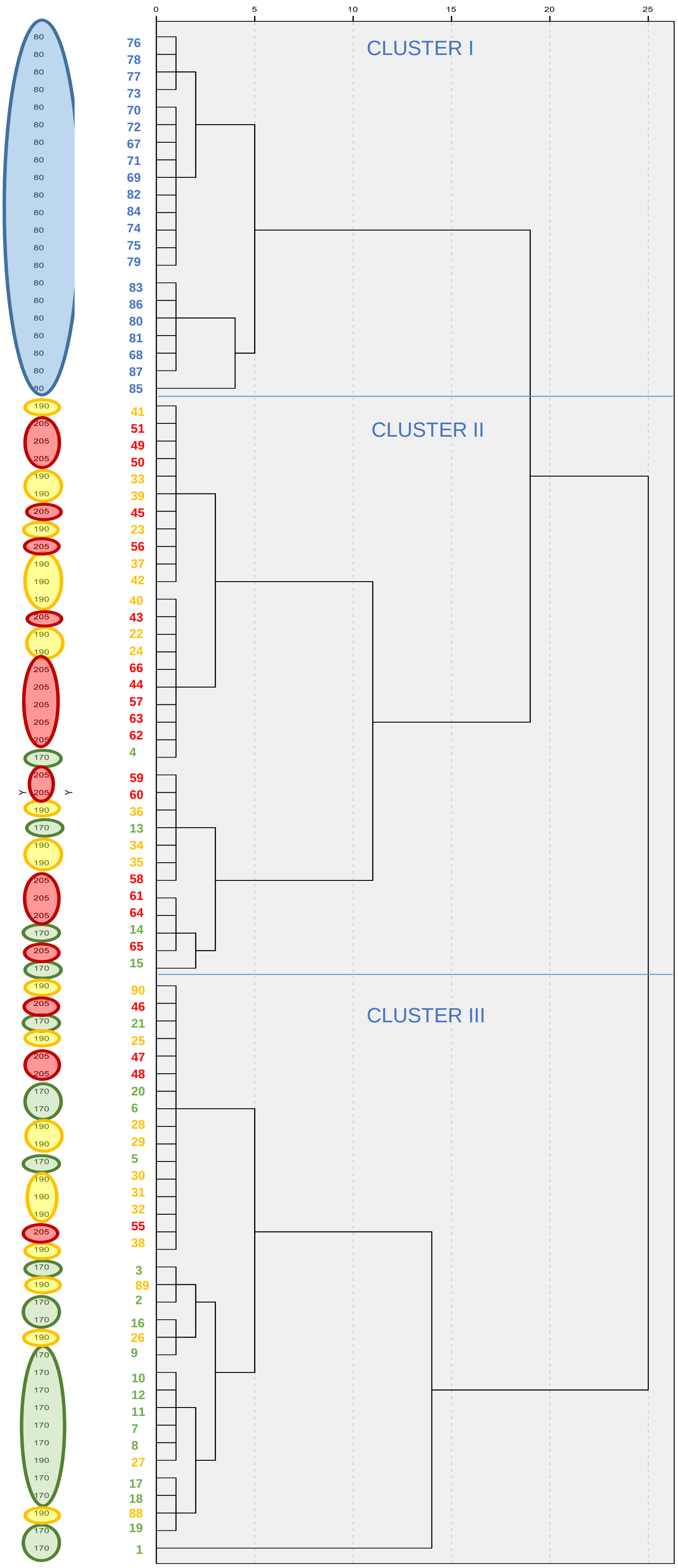

Figure S6. Hierarchical dendrogram based in principal component analysis of the 2nd derivate of the FTIR spectral region from 2300 to 2100 cm^{-1}. To clarify PCA results (3.1.2. and Figure 6e), coefficient scores obtained by PCA analyses were arranged in a hierarchical dendrogram**.** Similar to Figure 6e, a homogeneous cluster can be observed composed by 80ºC series (cluster I). Rest of the experiments are distributed in two clusters, one of them presents most of the 205 ºC experiments (cluster II), similarity to Figure 6e. Colored ellipses are used to indicate the experimental series: green represented samples from the 170 ºC series, yellow 190 ºC series, red 205 ºC series and blue 80 ºC series. The numeration of the experiments correspond with the Table S1. The samples in boxes are samples synthetized using equivalent reactions time based on the specifications of the microwave manufacture.

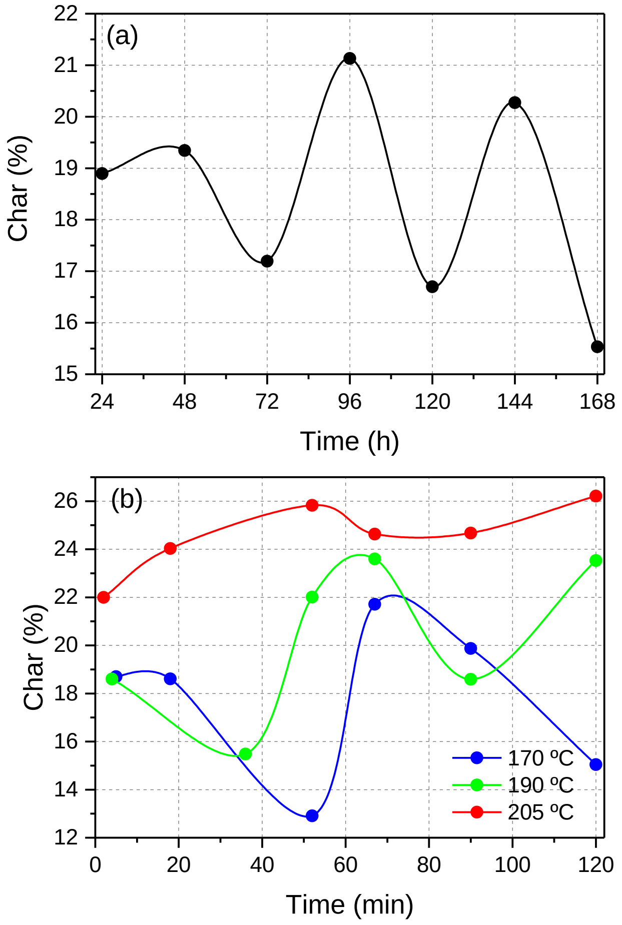

Figure S7. Evolution of the char (%) after heating of the samples at 1000 ºC as function of the reaction time. (**a**) 80 ºC series; (**b**) MWR series.

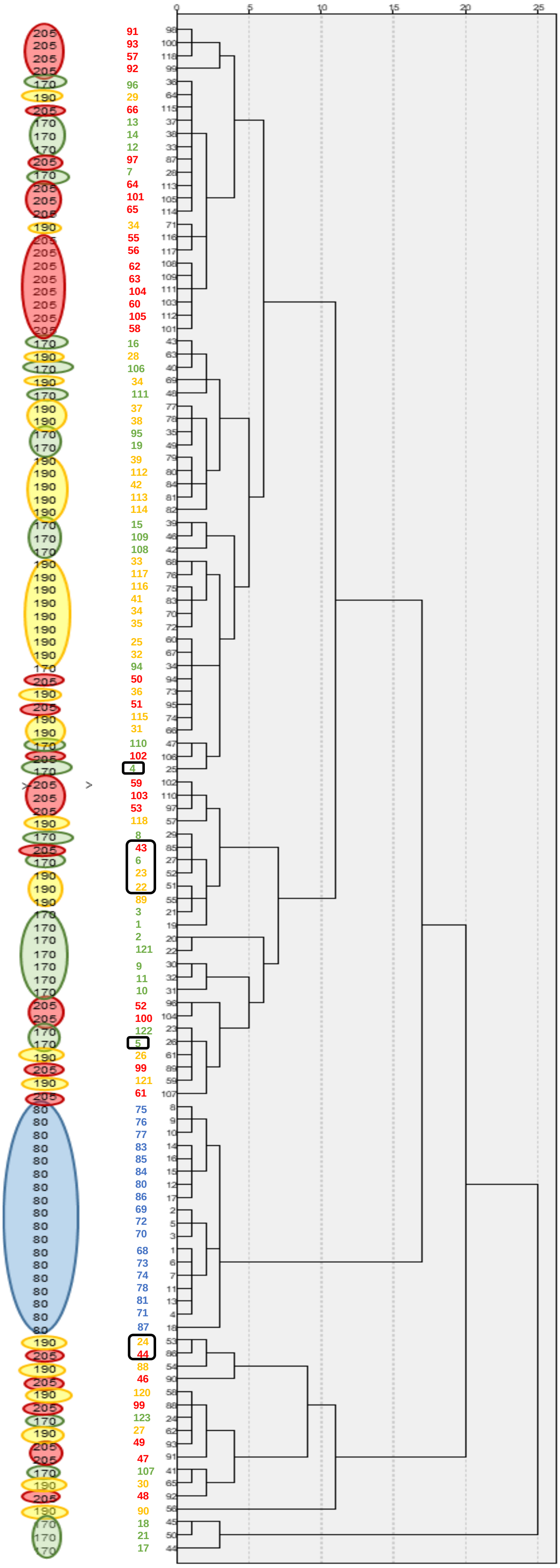

Figure S8. Hierarchical dendrogram based in principal component analysis of the UV-vis spectra. To clarify PCA results (3.2. section and Figure 12), coefficient scores obtained by PCA analyses were arranged in a hierarchical dendrogram. Similar to Figure 12, a homogeneous cluster can be observed composed by 80 ºC series. The numeration of the experiments correspond with the Table S1. The samples in boxes are samples synthetized using equivalent reactions time based on the specifications of the microwave manufactory.

REFERENCES

Fernández, A.; Ruiz-Bermejo, M.; de la Fuente, J. L. Modelling the kinetic and structural properties evolution of a versatile reaction: The aqueos HCN polymerization. Phys. Chem. Chem. Phys. **2018**, *20*, 17353-17366.

Mas, I.; de la Fuente, José L.; Ruiz-Bermejo, M. Temperature effect on aqueous NH_4CN polymerization: Relationship between kinetic behaviour and structural properties. Eur. Polym. J. **2020,** *132*, 109719.

Ruiz-Bermejo, M.; de la Fuente, J. L.; Carretero-González, J.; Luis García-Fernández, L.; Aguilar, M. R. A comparative study of HCN polymers synthesized from NH_4CN or DAMN polymerization in aqueous media: New perspectives for prebiotic chemistry and material science. Chem. Eur. J. **2019**, *25*, 11437-11455.
